# Dynamic gill and mucus microbiomes during a gill disease episode in farmed Atlantic salmon

**DOI:** 10.1038/s41598-022-17008-2

**Published:** 2022-10-06

**Authors:** Victor B. Birlanga, Grace McCormack, Umer Z. Ijaz, Eugene MacCarthy, Cindy Smith, Gavin Collins

**Affiliations:** 1 Microbiology, School of Natural Sciences, National University of Ireland Galway, University Road, Galway, H91 TK33 Ireland; 2 School of Natural Sciences, National University of Ireland Galway, University Road, Galway, H91 TK33 Ireland; 3 Ryan Institute, National University of Ireland Galway, University Road, Galway, H91 TK33 Ireland; 4 Infrastructure and Environment, School of Engineering, University of Glasgow, Rankine Building, Oakfield Avenue, Glasgow, G12 8LT UK; 5 Institute of Science, Technology and Medicine, Galway-Mayo Institute of Technology, Galway, H91 T8NW Ireland

**Keywords:** Marine microbiology, Microbiology, Bacteria, Microbial communities, Environmental microbiology

## Abstract

Amoebic gill disease (AGD) and complex gill disease (CGD) are recurrent gill disorders in Atlantic salmon, resulting in significant aquaculture losses. The role of gill microbiomes in gill disease development is unclear. We undertook a longitudinal study to characterise the gill tissue and gill mucus microbiomes of farmed Atlantic salmon before, and during, a gill disease episode. Using a newly optimised DNA extraction protocol, we sequenced rRNA genes from microbiomes of gill samples taken from 105 individual salmon on a farm, over a summer season. The AGD aetiological agent, *Neoparamoeba perurans*, was PCR-quantified targeting 18S rRNA genes. Similar analyses were carried out on mucus samples. Mucus scrapings were suitable, non-lethal substitutes for characterisation of the gill prokaryotic community in this study. Gill tissue and gill mucus microbiomes changed during the campaign, correlating with *N. perurans* concentrations. Time explained 35% of the gill tissue and gill mucus microbiome variance, while *N. perurans* concentrations explained 5%. Genera including *Dyadobacter, Shewanella* and *Pedobacter* were maximally abundant in gill and mucus samples at the timepoint prior to the the detection of gill disorder signs, at T3. *Shewanella* was significantly more abundant before than during the gill disease episode, and we suggest this genus could be considered in future studies addressing relationships between gill disease and the gill microbiome.

## Introduction

Global fish production was 179 million tonnes in 2018, of which aquaculture represented 46%^1^ and approximately 2.4 million tonnes were production of Atlantic salmon (*Salmo salar* L.)^1^. The average annual growth of worldwide fish consumption was 3.2% between 1961 and 2016, which was twice the rate of human population growth and significantly more than the growth in meat consumption^2^. Aquaculture production is expected to expand from 179 million tons in 2018 to 204 million tons by 2030^1^, representing over 50% of total fish-derived protein.

However, the aquaculture industry faces several production challenges, including those related to infectious diseases. In particular, rates of gill disorders in salmon have increased in many countries^3^, resulting in significant burdens on the sector due to lower growth rates, and increased susceptibility to other pathogenic agents and mortality. Amoebic gill disease (AGD) and complex gill disease (CGD) are currently two of the most important among the gill pathologies^4–6^, resulting in significant economic impact for the industry^7,8^.

The most prevalent signs of AGD include uncontrolled proliferation of epithelial cells from fish gills, overproduction of gill mucus, respiratory distress, and, in some cases, fish death, mainly due to reduced gas exchange^9^. The aetiological agent of AGD is the free-living amoeba *Neoparamoeba perurans*^10^. However, CGD is the result of a combination between various gill disorders (including AGD), without a principal pathogen^6^. So far, the most common means to reduce AGD and CGD is by controlling the concentration of the pathogens on gills, with the application of freshwater baths^4,8^ or hydrogen peroxide^4,11^. The clearest gross sign of *N. perurans* infection is the presence of white patches on gills, allowing classification of gill health using a gill score describing the extent of the patches and, thus, the disease^12^. In addition, a suite of molecular diagnostic tools is now available^10,13^, which allows *N. perurans* to be directly targeted from community DNA from water or gill samples. However, and despite inherent sensitivity and specificity, quantitative-PCR assays targeting *N. perurans* are only useful in confirming colonisation of the gill by the amoeba, and cannot be used to determine vulnerability to AGD prior to *N. perurans* colonisation. Indeed, our understanding of several aspects of AGD and CGD are incomplete, including regarding the density of parasites and pathogens required to provoke signs; the involvement of other microorganisms, such as viruses, bacteria and zooplankton; and environmental parameters^4,6,14,15^.

It is well documented that the microbiome of plant and animal tissues plays important potential roles in several diseases of various macro-eukaryotes, variously affecting the severity of signs^16–20^. Indeed, fish are known to harbour natural microflora^21^ that play a role in host defenses against pathogens^4^. The impact of AGD and CGD on the microbiome of the salmon gill, and vice versa*,* is unclear. To date, some studies have reported on AGD, and associated bacteria^22–28^, identifying the presence of various bacterial taxa only in AGD-affected fish. However, those studies were either characterised by the relatively low-throughput nature of the culture-independent approaches, or by the inherent limitations of laboratory trials with little variation of environmental conditions and reduced diversity of free-living microorganisms in the surrounding water. Thus, our understanding of the role of the gill microbiome in gill health of seawater-farmed salmon, and how it responds during gill diseases outbreaks, is limited^6,8^.

We undertook a longitudinal study with the main objectives of characterising the gill prokaryotic microbiome of a cohort of farmed Atlantic salmon (*Salmo salar* L.) and quantifying predominant genera, before, and during, a gill disease episode.

To achieve this, we also pursued two methodological objectives. First, we needed to develop an approach to reliably sample the gill microbiome. Although previous studies have applied molecular tools to study, gills^14,26–33^, typically only partial gills (sometimes less than 10% total gill arch weight) have been used as samples. Equally, there are few data available on the efficiency of gill microbiome DNA recovery techniques. Thus, we first undertook to optimise the extraction based on sampling of entire gill arches. In that way we avoided extrapolation of results from partial gills to the whole gill microbiome, or under-sampling and under-representation of taxa in the microbiome.

Finally, as gill sampling is inherently invasive, a non-lethal and less invasive sampling approach would provide the basis for more attractive monitoring tools. Mucus sampling, typically as a gill swab, has been widely used in characterising the gill microbiome; but this non-lethal sampling approach has not been demonstrated to be a representative alternative to sample the entire gill arch. Thus, our second methodological objective was to test the efficacy of sampling mucus as a suitable alternative source to gill tissue for microbiome analyses.

## Results

### Representative method for nucleic acids extraction from fish gills

**Figure 1:**
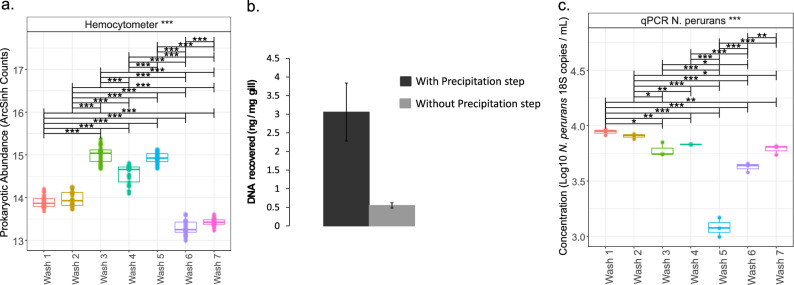
ANOVA of pair-wise comparison of extrinsic metadata considered in this study, and after applying appropriate normalization (where necessary). Lines connect two categories where the differences were significant with *(*P* < 0.05), **(*P* < 0.01), or ***(*P* < 0.001). (**a**) ArcSinh normalisation was used for prokaryote cells from each wash); (**b**) comparison of DNA yield from extractions with and without isopropanol and acetic acid precipitation (replicate treatments); (**c**) qPCR data from each wash transformed using Log10 of the original results for *N. perurans*. Figure titles with *** refer to analyses that included significant differences.

The number of prokaryotic cells in the liquid phase increased across the first five of the successive washes. However, significantly fewer cells were counted after the sixth and seventh washes, suggesting that five such washes sufficiently recovered the majority of the prokaryotic cells on the gills (Fig. 1). Alpha and beta-diversity (supported by PERMANOVA *P* > 0.05) were not significantly different between each wash, similarly resolving the prokaryotic communities (Fig. S1; i.e. comparing washes). Rarefaction curves (Fig. S2) indicated DNA sequencing was close to the saturation limit (or plateau) in each of the separate washes after host DNA removal (~ 75% of the reads).

The second step of the DNA extraction procedure (i.e. the precipitation step) resulted in a four-fold increase in DNA recovery (Fig. 1). The average DNA recovery from gill samples was 3 ng DNA/mg wet gill.

Quantitative PCR assays indicated abundant amoebae along the washes (Fig. 1). Although the concentration of *N. perurans* 18S rRNA genes was relatively low after the fifth wash, the concentration was higher again after the sixth and seventh washes.

### Fish features and the onset of gill disease

**Figure 2:**
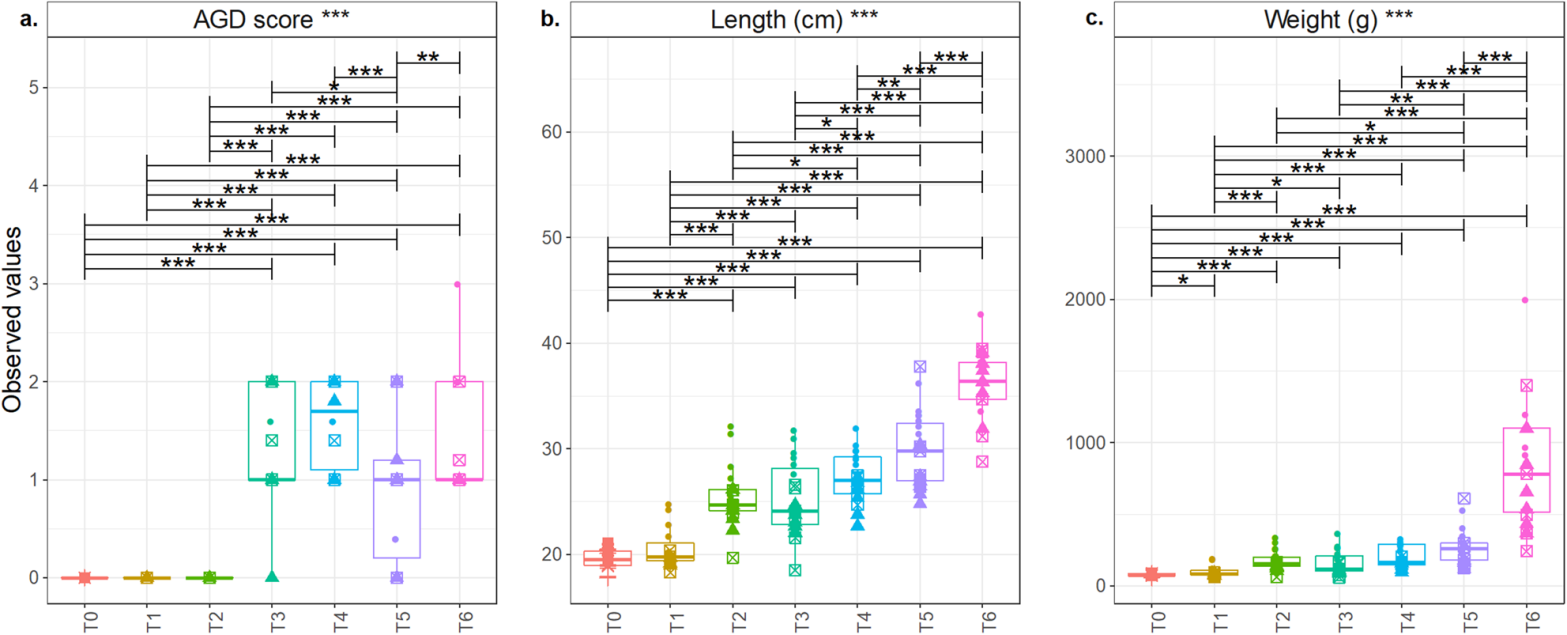
ANOVA of pair-wise comparison of extrinsic metadata considered in this study, and after applying appropriate normalization (where necessary). Lines connect two categories where the differences were significant with *(*P* < 0.05), **(*P* < 0.01), or ***(*P* < 0.001). Mean (**a**) Gill score, (**b**) length (cm), and (**c**) weight (g) at each of the timepoints. Figure titles with *** refer to analyses that included significant differences.

The length and weight of the salmon cohort increased over the sampling campaign (Fig. 2), and significantly so betweenT5 and the final sampling point (T6). The mean length and weight at T0 were 19.5 cm and 72.6 g, respectively, and at T6 were 36.4 cm and 847.3 g, respectively (Table S4).

**Figure 3:**
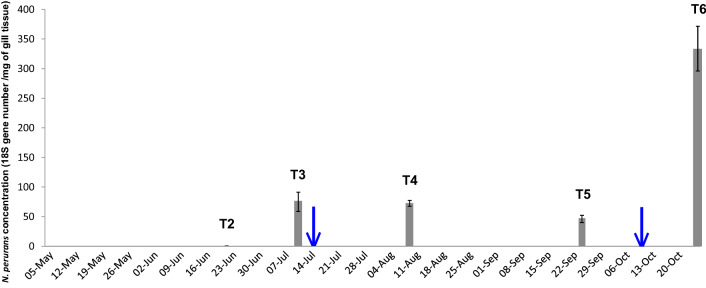
Concentrations of *N. perurans* 18S rRNA genes in DNA extractions from salmon gills measured by qPCR assays along the sampling campaign (from May until October 2017). Blue arrows mark the time when the fish farm treated salmon with freshwater baths.

Based on gross scoring, no clear signs of gill disease were apparent until T3 (Fig. 2). The condition factor noticeably disimproved between T3 and T5, improving later by T6, which may have been associated with other environmental conditions not monitored by the study. *N. perurans* was detected in two samples at T2 but only after 39 PCR amplification cycles (Fig. 3), and close to the limit of detection of the assay (Ct, 40.13)^13^. Based on this, and the associated gill scores, both samples were considered AGD-negative. The first gross signs on gills were detected between T2 and T3. Considering this, the farm decided to apply freshwater baths to the salmon stocks three weeks before T4, and two weeks before T6 (Table S4). All salmon from the same cage were freshwater bathed (salinity, 0.1–0.6‰) at the same time for three hours.

Our case definition of an AGD-affected fish comprised those salmon with a gill score of ≥ 1 and a positive *N. perurans* qPCR result. Previous studies have used only gill scoring and qPCR results to define AGD cases^34,35^. Thus, samples from T3, T4, T5, and T6 were considered to be AGD-affected but are assumed in this study to have CGD. Histological analyses of gills was not used and so we mainly describe a gill disease rather than AGD or CGD.

### Temporal microbiome development of the salmon gill microbiome

After the removal of host DNA reads, the number of reads from all samples from the longitudinal gill microbiome study was characterised by: 1st quantile: 6063; median: 27,684; mean: 41,221; 3rd quantile: 65,769; max: 210,382. Reads from samples from the gill and mucus comparison were summaried as: 1st quantile: 4774; median: 22,601; mean: 43,180; 3rd quantile: 71,340; max: 210,382. During sample processing, samples with fewer than 2000 total reads were excluded, and thus six samples (four gill and two mucus samples) were removed before final analyses, resulting in a total of 129 samples (101 gill and 28 mucus samples). In the final analysis, a total of 3834 clean OTUs from the *n* = 129 samples were included.

**Figure 4:**
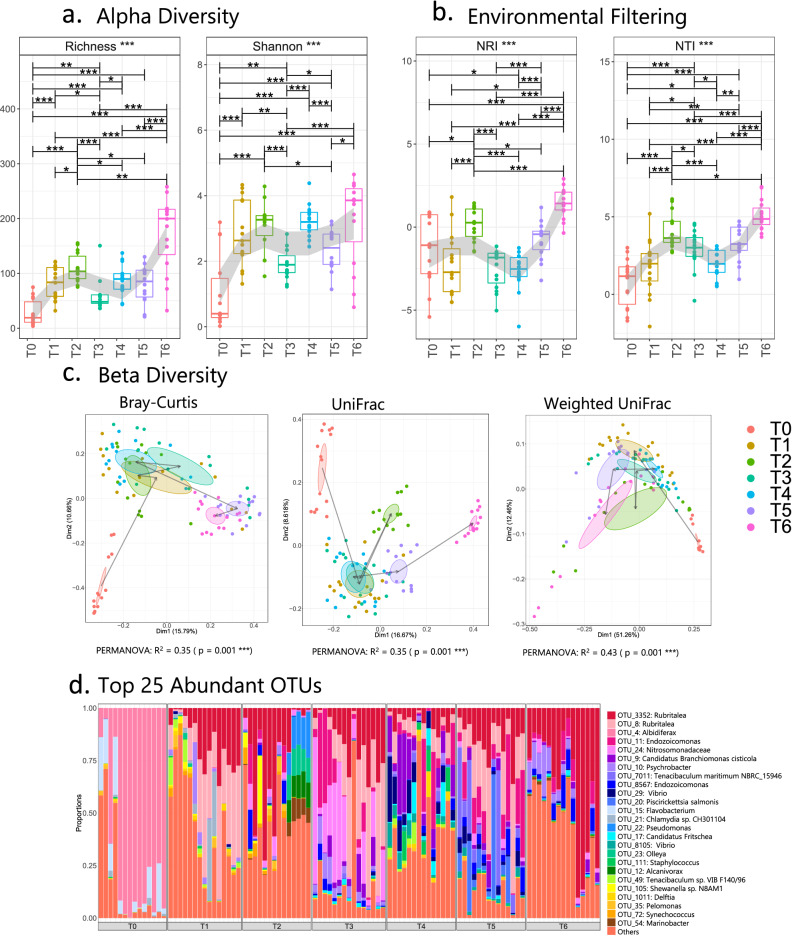
Microbial diversity and community structure from temporal gill analyses. (**a**–**c**) represent alpha diversity, environmental filtering, and beta diversity indices, respectively. In (**a**) and (**b**), the regions have been shaded using LOESS smoothing in geom_smooth() when plotting using R’sggplot(). In (**c**), ellipses are drawn at 95% confidence interval of standard error with lines from T0 to T6 plotted at the mean of the ordination values at each timepoint. (**d**) shows community structure based on relative abundance of the top 25 most abundant OTUs from across each timepoint, where ‘others’ refers to all OTUs not included in the top 25; lines for figures (**a**) and (**b**) connect two categories where the differences were significant (ANOVA) with *(*P* < 0.05), **(*P* < 0.01), or ***(*P* < 0.001). Figure titles with *** refer to analyses that included significant differences.

The richness and Shannon index of the prokaryotic community on gills increased on transfer to seawater and at T2 (Fig. 4a, b). At T3, following the first observation of gross signs of gill disease, and the detection of *N. perurans*, the microbiome exhibited significantly reduced diversity and lower NRI (environmental filtering) compared with T2 and with T4–T6.

Beta-diversity analyses (Fig. 4c) using the Bray–Curtis metric indicated the gill microbiome of pre-smolted salmon was completely different to the community on gills of post-smolted salmon. This was consistent with beta-diversity using UniFrac and Weighted UniFrac metrics. Once all salmon were transferred to seawater, the bacterial community did not sharply change until the first gill disease signs were detected by T3. The gill microbiomes on T5 and T6 clustered separately from the earlier samples.

The genus *Rubritalea* was found to be present consistently in microbiomes from gill samples through the whole sampling campaign, except at T0 (Fig. 4d). The gill microbiome from pre-smolted salmon was predominantly composed of bacteria from the genus *Albidiferax* and *Flavobacterium.* However, after being transferred to seawater and during the rest of the sampling period, more prokaryotes, including *Shewanella*, *Synechococcus, Marinobacter*, *Endozoicomonas*, *Tenacibaculum*, *Pelomonas*, *Alcanivorax* and *Staphylococcus,* were abundant on gills. In general, microbiomes appeared to somewhat group according to cages, although we did not investigate this further. A less diverse community was present on gills during the gill disease episodes, when bacteria from *Vibrio*, *Pseudomonas* or *Psychrobacter* appeared more abundant. Some other genera were found on infected gills, including *Ca*. Branchiomonas cisticola, *Tenacibaculum maritimum*, *Piscirickettsia salmonis*, *Piscichlamydia* sp., *Psychroserpens* sp. and *Ca*. Fritschea sp. Those are known as putative pathogens that have previously been detected in diseased Atlantic salmon (Table S1), and some are associated with CGD.

Based on sPLS-DA analyses of discriminating genera (Fig. S4e), gill samples from the same timepoint clustered together, except those from T1 and T2. Some genera (e.g., *Albidiferax*, *Altererythrobacter*, *Pedobacter*, *Dyadobacter* and *Shewanella*) were abundant immediately prior to the gill disease onset at T2, almost two weeks before the first gross signs were detected. These genera were not observed (or not abundant) at any of the remaining timepoints.

### Mucus scraping to approximate gill microbiomes

**Figure 5:**
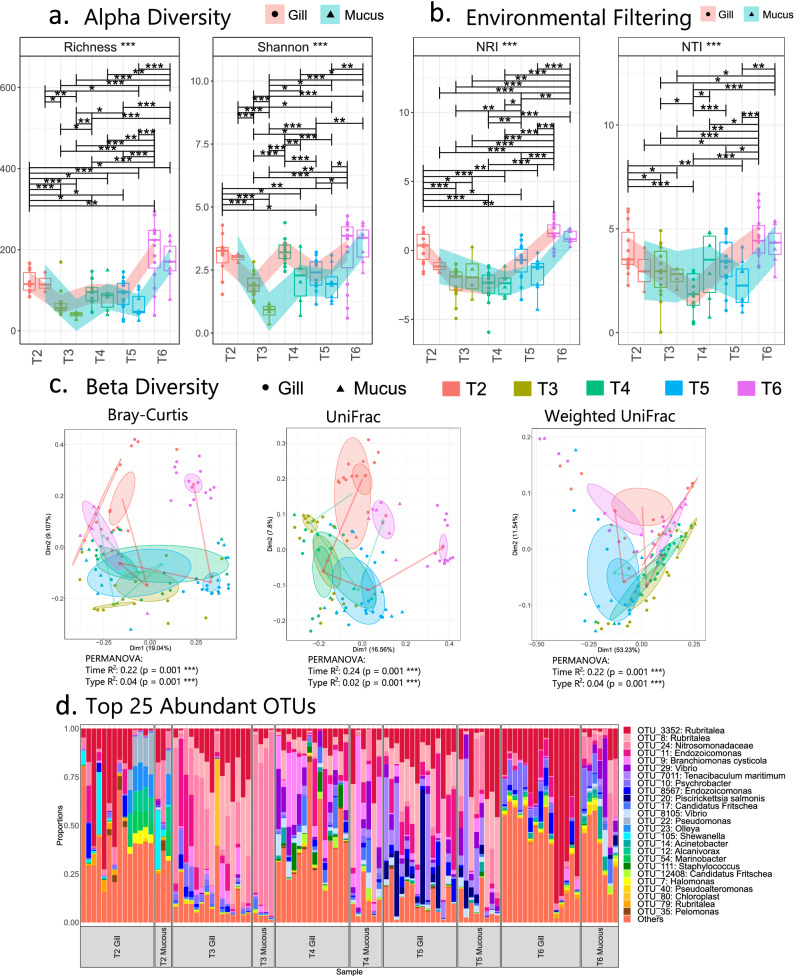
Microbial diversity and community structure from for cross-sectional ‘gill vs mucus’ analyses. (**a**–**c**) represent alpha diversity, environmental filtering, and beta diversity indices, respectively. In (**a**) and (**b**), that LOESS smoothing was performed separately on gill and mucus samples, and PERMANOVA now considers both *Time* and *Type* parameters. In (**c**), ellipses are drawn at 95% confidence interval of standard error with lines from T2 to T6 plotted at the mean of the ordination values. (**d**) Shows community structure based on relative abundance of the top25 most abundant OTUs; lines for figures (**a**) and (**b**) connect two categories where the differences were significant (ANOVA) with *(*P* < 0.05), **(*P* < 0.01), or ***(*P* < 0.001). Figure titles with *** refer to analyses that included significant differences.

The alpha-diversity profiles of gill microbiomes were similar to the mucus microbiomes before the gill disorder onset (Fig. 5a). Shannon diversity indices of gill and mucus samples deviated significantly after the first detection of signs (T3), and gill mucus microbiomes were significantly less balanced than gill tissue microbiomes at both T3 and T4.

No distinguishable patterns were apparent in gill or mucus samples using environmental filtering or beta-diversity analyses (Fig. 5b, c). *Rubritalea*, which were apparent in gill samples (previous section), were also found in all corresponding mucus samples (Fig. 5d) at the same timepoints. Microbiome variability in both gill and mucus samples was significantly affected by both time (23%) and sample type (mucus or gill; 3%), and mucus samples appeared to provide at least a partial characterisation of corresponding gill microbiomes (Fig. 5c).

### Sources of variation in N. perurans concentration

The concentration of *N. perurans* DNA was strongly related to the sample type (gill or mucus) and strongly negatively associated with water temperature, which was 12.2–16.0 ºC over the campaign (Supplementary Data: Environmental Parameters and Table S3). On the other hand, it was significantly more likely to detect *N. perurans* from gill mucus than from gill tissue samples. No relationship was apparent between fish features (i.e. weight and length, or gill score) and *N. perurans* concentration. Negative associations were observed between the concentration of *N. perurans* and salinity (ranging from 28 to 34‰), oxygen concentrations (ranging from 86.5 to 106.5%) and water clarity (ranging from 4 to 6 m).

### Associations between N. perurans, and environmental factors, and the gill microbiome

A subset of nine OTUs explained 92% of dissimilarity (Bray–Curtis distance) between gill samples (Table S2). Time explained 35% of variability among all gill OTUs but only 20% of variability in the subset. Similarly, *N. perurans* concentration explained 5% of variability across all OTUs but only 3.7% in those nine OTUs.

Observing only data from gill microbiomes, only one group —*Stenotrophomonas* sp.—was correlated (negatively) with any of the environmental metadata (water clarity) (Table S2).

Considering both mucus and gill samples, a sub-set of 12 OTUs accounted for 91% of all dissimilarities across all OTUs (Table S2). In this case, sample type (mucus or gill) was also considered in PERMANOVA analyses. Time explained 22% of variability amongst all OTUs but only 20% of variability in this sub-set. *N. perurans* concentration could explain 2.4% of variability in the sub-set.

Again, considering mucus and gill data collectively, four OTUs from both gill and mucus microbiomes correlated with environmental metadata, of which only two OTUs correlated with fish features. One OUT—*Gardnerella* sp.—positively correlated in the gill dataset with gill scores, but negatively with fish weight in both gill and mucus datasets.

MINT analysis of gill and mucus samples found *Altererythrobacter*, *Dyadobacter*, *Shewanella* and *Pedobacter* were maximally abundant on gills and in mucus samples at T2 (Fig. S5), when *N. perurans* DNA was below the reliable limit of detection by qPCR (Fig. 3).

## Discussion

### Advantages of the extraction protocol and scope for improvement

Representativeness in sampling has been widely addressed across different fields^36^, including in contaminants degradation^37^, macroorganism ecology^38^ and microbial ecology^39^, since under-sampling reduces the reliability of observed patterns. Microbial analyses from field samples normally indicate high spatial and temporal heterogeneity, making representativeness critical in microbial ecology. Previous studies, such as Steinum et al*.*^14^, Schmidt et al.^30^, Slinger et al.^26,31^, Botwright et al.^28^, Gunnarsson et al.^32^ and Clinton et al.^33^ have characterised bacterial communities from partial gills, but the approach may underestimate diversity in the microbiome. Commonly used nucleic acid extraction kits limit the volume, or mass, of sample in extraction preparations to normally between 30 and 100 mg, which would preclude the use of entire gill arches (which typically exceed 100 mg).

Although it is also possible to extract DNA and RNA from whole gills using in-house purification protocols^29,40^, commercially available isolation kits offer more reproducibility and, usually, user-friendly workflows. In addition, nucleic acid extraction protocols commonly incorporate a tissue disintegration step with bead-beating^29^, resulting in high relative concentrations of host DNA in final extracts. Host DNA competes with microbiome DNA for PCR amplification, and sequencing depth, obscuring patterns in microbial community analyses^41^. Thus, a step to allow for isolation, and concentration, of microbial cells from whole gill arches is desirable. The DNA and RNA extraction protocol presented in this study included this step prior to the use of a commercially available extraction kit. This ensured DNA extractions comprised the nucleic acids from a representative part of the microbiome on gills, by extracting DNA from five pooled gill washes including the precipitation step (which concentrated all free DNA and floating cells in a smaller volume). Although additional *N. perurans* could be detected after the fifth wash, the first step of the protocol was established as a series of *five* washes in order to keep the DNA extraction as simple and cheap as possible. In addition, this study was focused on the prokaryotic microbiome in AGD rather than only on the *N. perurans* concentration on gills.

Salmon DNA was present in extractions and roughly three quarters of reads were from host genes. Nevertheless, after removing host DNA from the analyses, the saturation of the rarefaction curves from bacterial sequences indicated no significant loss of measured bacterial diversity despite host contamination. Nonetheless, competition from high concentrations of host DNA could inhibit amplification of prokaryotic targets. Further modifications to the proposed DNA extraction procedure could be considered, including to deplete as much host DNA as possible using, for instance, blocking-primers^42^, propidium monoazide^41^ or methylation-based enrichments^43^.

### Temporal microbiome development

It was reasonable to consider that the gill disease started between T2 (June) and T3 (July) as the first gross signs were detected in early July and continued until T6. *N. perurans* concentration and gill scoring during this period supports this (Figs. 2, 3). It was possible that the amount of *N. perurans* DNA recovered was limited by the detachment of amoeba from gills due to the anaesthesia (MS-222)^44^, anda larger sampling area could allow recovery of more amoeba from the gills^45^. However, the salmon cohort did not show any gross sign of gill disease until T3 (Fig. 2), suggesting that the stock was not affected by the gill disease until then. Hence, even if *N. perurans* already colonised those salmon at T1 or T2, we cannot conclude that the gill disorder started until T3.

Some bacterial taxa, for example *Rubritalea*, *Endozoicomonas*, *Nitrosomonas*, C. *Branchiomonas cysticola*, *Psychrobacter*, *Tenacibaculum*, *Piscirickettsia*, *Flavobacterium*, *Shewanella*, *Synechococcus, Pseudomonas*, C. *Fritschea*, *Staphylococcus*, *Alcanivorax* and *Winogradskyella*, found in the microbiome from gill and mucus samples, were already described in previous gill microbiome studies (Table S1). However, this was the first study to detect the genus *Rubritalea* from Atlantic salmon gill samples in the field.

Previous studies suggested that some bacteria (*Psychroserpens*, *Winogradskyella* and *Tenacibaculum dicentrarchi*) could have a connection with the AGD on Atlantic salmon gills^22–24,26^. However, *Winogradskyella* was not apparently differentially abundant on gill samples before and during the disease episode, suggesting it may not be associated with the onset, though perhaps with the severity, of AGD or CGD^24^. Species from the *Tenacibaculum* genus (*Tenacibaculum maritimum*) and *Psychroserpens* were more abundant during AGD, supporting the idea that both could be associated with affected gills^22,26,46^. Other reports observed a close association between *N. perurans* and *Vibrio* species cultured in vitro^25^. In this study, species from the genus *Vibrio* were much more abundant during the disease episode in gill and mucus samples (Fig. 5d), when the *N. perurans* concentration increased (Fig. 3), adding evidence supporting a possible connection, but now from field samples.

Other bacteria associated with various gill diseases were prominent on infected gills. Those included: *Ca*. Branchiomonas cisticola, *Piscirickettsia salmonis*, *Piscichlamydia* sp. and *Ca*. Fritschea sp. (Table S1), the presence of which on salmon gills during the episode may have contributed to gill damage, increasing the severity of the already existing gill lesions, as was suggested previously by Steinum et al*.*^14^. It is desirable that future studies aiming to study any aspect of the gill microbiome in relation with a gill disease include further understand of the overall salmon health status. Due to circumstances beyond the authors’ control, it was not possible to continue sampling after T6, when gill scores and qPCR data indicated the salmon population was still affected by a gill disease.

Principally, the study provided a temporal profile of the microbiome on Atlantic salmon gills by focusing emphasis on microbial diversity metrics. Prokaryotic richness and community balance on gills of pre-smolted salmon were the lowest across the dataset. The environmental filtering analysis in our study, which considers phylogenetic clustering as a cue for environmental pressure, suggested that the prokaryotic microbiome on the pre-smolted salmon gills may have inherent stochasticity, driven by competition with other taxa. The gill microbiome may follow a similar trajectory to the gut microbiome^19,47^, whereby an initial phase characterised by low diversity in the microbial community transitions, with changes in salmon lifestyle, toward a richer and balanced community. After being transferred to seawater, there was a marked change in prokaryotic diversity on salmon gills, preserving balance and richness. The shifting salinity from freshwater to seawater, however, can change the gill microbiome, as described previously for skin microbiomes^48^. Future studies should incorporate frequent sampling, so that gill microbiomes stabilise before after the onset of gill disease.

The alpha diversity of the prokaryotic gill microbiome increased after being transferred to seawater, as was also observed by Llewellyn et al*.*^19^. In addition, the environmental filtering analysis suggested the main environmental pressure was around T2 (which was also supported by beta-diversity analyses). However, one week after the salmon showed the first gross signs of a gill disease (T3), the environmental pressure was eased and the structure of the microbial communities appeared less deterministic, driven instead by a competitive exclusion principle (the ecological principle whereby community assembly is unhindered, and is driven by competition amongst taxa). Those trends were previously observed in the literature, albeit in connection with a *Helicobacter pylori* gastric infection in human and its gut microbiome^49^. At the same time, *N. perurans* concentrations, determined by qPCR profiles, were high at T3 when compared with the *N. perurans* presence at T2. Thus, we suggest that changes in the microbial community structure were partially due to the influence of AGD. Slinger et al*.*^26^ demonstrated that the alpha-diversity (measured like Shannon diversity) from AGD-affected gills was significantly lower than gills from AGD-naïve salmon. Although the present study could not include negative controls, the same correlation between healthy gills and gills affected by gill disease was observed between same-cohort fish from T2 and T3 (Fig. 4). Nonetheless, time accounted for a significant proportion of the variance in the community and so T2 cannot be used to control for T3 data.

### Comparing gill and mucus microbiomes

None of the predominant bacterial taxa were found to be unique to either of the sample types (Figs. 4, 5). This was not unexpected, as the DNA extraction from gill samples could be considered as a DNA co-extraction from both mucus and gills. The mucus on gills is likely to detach from the gill to the wash solution during the DNA extraction protocol; hence, communities can intermingle with more homogenised microbial community structures for the two samples types. Thus, our null hypothesis was that there is a degree of similarity in the bacterial communities from mucus and gill samples. However, when we performed diversity analysis, we found significant differences in Shannon entropy between gill and mucus samples at T3 and T4.

Although some differences between gill and mucus samples were found, the similarity of the sample types with respect to prokaryotic diversity (Fig. 5) suggests mucus scrapings appear effective for partial characterisation of the whole-gill prokaryotic microbiome. This initial evidence requires further exploration in future studies.

*N. perurans* concentration was significantly positively correlated with mucus microbiomes in every subset regression model (Table S3). This confirms it was more likely to quantify more *N. perurans* in the mucus than in the gill samples, making non-lethal mucus scraping more reliable in targeting *N. perurans* from salmon gills. This would be supported by the study from Downes et al.^45^, which found gill swabs to show a higher proportion of AGD-positive results than filament biopsies from gills.

Furthermore, three bacterial genera (*Shewanella* sp., *Dyadobacter* sp. and *Pedobacter* sp.) were most relatively abundant at T2, before the gross signs in gill samples at T3 (Fig. S5). The genera were previously found on intestine, skin, eggs and gill microbiomes from various fish species (Table S1). Of these, *Shewanella* sp. were amongst the most abundant OTUs in this study and were widely detected in skin and gill microbiomes reported in the literature^14,50,51^. Since its relative abundance significantly changed after the onset of gill disease , we suggest that *Shewanella* sp. could be of interest in further investigations using co-cultures with other relevant organisms associated with gill diseases.

### Sources of variation of the microbiome on salmon gills

Microbiome subset analyses of gill and mucus samples found several positive and negative correlations between the most variable part of the prokaryotic community and the environmental factors examined (Table S2). Nevertheless, only one bacterial taxon correlated with fish features. *Gardnerella* sp. significantly, positively correlated with the gill score but negatively correlated with salmon weight.

Several intrinsic and extrinsic sources of variation shape microbial communities, from study design to environmental factors. Environmental parameters explained 31% of the variability in community structure (Table S2). *N. perurans* explained 5% of the variability in the whole prokaryotic community from gills and mucus. Thus, the connection between the development of AGD (*N. perurans* quantification) and the prokaryotic microbiome on farmed Atlantic salmon gills was significant, although the measured environmental parameters had a higher impact on the gill microbiome.

Furthermore, *N. perurans* concentration significantly correlated with subsets of the microbiome (Table S2). However, further studies would be required to better understand the relationships between specific gill bacteria and causative agents of gill diseases. Additional field studies characterising the gill microbiome in AGD or CGD-affected salmon from other cohorts and locations would be required to determine the extent of such relationships, and to compare with the present study. Laboratory trials to test the influence on gill disease of specific marker genera would also be necessary. Metagenomics of the prokaryotic community on AGD-affected gills, combined with *N. perurans* transcriptomics, would assist in further elucidating such relationships.

Most of the literature targeting the study of the gill microbiome from Atlantic salmon^14,19,23,24,26–28,30,31^ has been performed in laboratory conditions. This implies a little variation of environmental conditions and a reduced diversity of free-living microorganisms in the surrounding water. In consequence, these studies obtained clearer conclusions, but were less representative of conditions in fish farms than studies sampling on them. On the other hand, studies that aimed to describe the gill microbiome from Atlantic salmon sampling in fish farms^19,22^ are more representative of the real conditions that those salmon experience. However, the high variability of environment makes more difficult to have clear conclusions about correlations. In conclusion, the combination of results from laboratory and field trials would allow for more complete understanding of gill disorders and the gill microbiome.

## Conclusions

In this study, a new procedure was optimised to representatively recover DNA from the microbiome of fish gills. Applying this optimised procedure, we were able to determine that, after the onset of a gill disease, the prokaryotic community on salmon gills shifted toward lower diversity and less balance. In addition, the tentative relationship between *N. perurans* concentrations and the prokaryotic microbiome from gill and mucus samples was suggested.

Whilst the range of the field study precluded a more fine-grained analysis of cause and effect, as might have been possible in laboratory trials or with co-culture experiemnts, the large scope of the work provides a descriptive overview of the behaviour of the gill microbiomes. The techniques applied to the collection and analysis of data will be helpful pointers for other investigators.

Three different genera were found to be maximally relatively abundant on gills and mucus samples before the gross signs of disease appeared. Among them, *Shewanella*, was in the top 25 most abundant OTUs, indicating this genus could be of interest to future studies aiming to better understand relationships between the gill microbiome and gill diseases. Bacterial communities from gill and mucus samples from this study presented very close similarities, suggesting that mucus scraping can be suitable for gill sampling for partial characterisation of the whole-gill prokaryotic community. Future research comparing these two sampling types can provide further evidence in this topic.

## Materials and methods

### Longitudinal Atlantic salmon gill microbiome sampling

**Figure 6:**
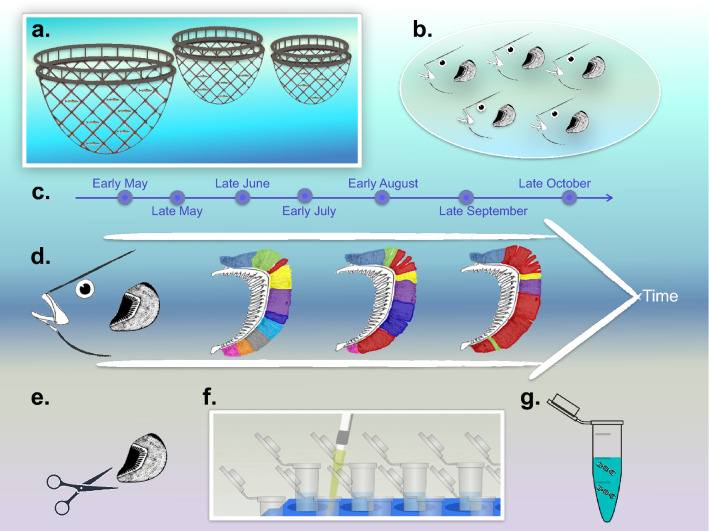
Overview of the sampling campaign and analytical approach. (**a**) Three cages were sampled to retrieve (**b**) five fish from each (n = 15) at each of (**c**) seven timepoints (n = 105) from May through October. (**d**) The hypothesis underpinning the study was that the amoebic gill disease (AGD) episode and concentration of *N. perurans* (red shading) would be associated with a shifting microbiome. The analytical approach included (**e**) sampling of gill and mucus samples, (**f**) a newly optimised DNA extraction protocol incorporating several successive washes, and (**g**) downstream analyses of nucleic acids. Figure titles with *** refer to analyses that included significant differences.

Salmon gill samples were collected from a fish farm in Bertraghboy Bay (western coast of Ireland; 53° 22′ 03.1″ N, 9° 51′ 47.5″ W), with the collaboration of MOWI Ireland. Sampling was started in Spring (T0, May 2017) using freshwater pre-smolts and followed the cohort for six months (T1, June–T6, October) in seawater cages (Fig. 6), during and after the summer months (Table S4), when AGD is more likely to appear (Oldham et al*.*^4^). At each of the seven timepoints (T0–T6), five salmon were sampled from each of three cages (Table S4), all of them under the same environmental and farming conditions. All salmon were sampled after attracting them to the edge of the cage with food and simply dip-netted. The fish were then over-anesthetised (tricaine methanosulfonate MS-222, 200 mg/L). The weight and length of each fish was measured, and the gill score was recorded by the same person in every timepoint, following the published guidelines^12^. The condition factor (K) of each fish was calculated using the given formula: K = 100 × [Weight (g) * (Length (cm)^3^)]^−1^^52^. Due to the observational, or descriptive, nature of the present study, we could not establish one salmon cohort in the fish farm as a negative control.

The gill microbiome was sampled as follows: first, the operculum was aseptically removed using sterile scissors; second, mucus was collected (only mucus samples from T2 to T6 were included in the study, as samples from T0 and T1 showed very low DNA yield after the extraction) from the first brachial arch of the left side of the fish by gently scraping using a sterile spatula, so only mucus was obtained. Mucus was sampled in this way from each of the five fish from the respective cages and pooled in 200 µl TE buffer (10 mM Tris–Cl, pH 7.5; 1 mM EDTA) in order to have enough DNA concentration after the extraction. When there was enough volume of sample, each of the three mucus pools (one from each cage) was then divided into two or three technical replicates for further processing. However all mucus samples from the same timepoint were considered as biological replicates in the statistical analyses. After the mucus sampling, the second brachial arch of the left side of the fish was excised using sterile scissors and forceps, and placed in respective sterile, labelled, tubes prior to flash freezing (*n* = 15).

Water temperature (ºC), salinity (‰), dissolved oxygen (%) and turbidity (Secchi depth, metres), were each measured daily throughout the sampling campaign at a depth of 5 m using a Conductivity-Temperature-Depth (CTD) measuring device (YSI 2030 device; ID: 12391).

### Optimisation of nucleic acids extraction from gills

To extract the DNA from gill samples, a three-step procedure was developed. First, 50 µl trypsin–EDTA (0.5% trypsin [w/v] in 6.8 mM EDTA; Sigma-Aldrich, Saint Louis, USA) were added along with 450 µl 0.5 M EDTA to a gill in a 2-ml microcentrifuge tube prior to sonication for 5 min (Kerry Ultrasonic Baths, model PUL-125). The gill was then removed to a fresh tube and the process was repeated multiple (seven) times. The number of washes required to optimally detach bacterial cells was determined based on the concentration of prokaryotic cells in the liquid phase from each successive wash measured using a hemocytometer (10 central squares [0.20 mm × 0.20 mm] were considered in each of three replicates for each wash). We could not determine the initial concentration of prokaryotic cells on the gills, which would be necessary to calculate the proportion of prokaryotic cells recovered.

Second, the liquid phase from each wash step was pooled in a 15-ml tube, and the gill was discarded. An equal volume of ice-cold isopropanol was added along with a 0.1 volume of 3 M sodium acetate to precipitate free DNA, which was then recovered with microbial cells by centrifuging for 30 min at > 8000 g (4 °C) to form three layers. The top and middle layers were discarded.

Third, an extraction kit (AllPrep DNA/RNA extraction kit, QIAGEN, Hilden, Germany) was used to recover nucleic acids from the bottom layer containing the pellet following the manufacturer’s instructions without any modifications.

To determine the efficacy of the second step in enhancing DNA extraction efficiency, DNA recovery was compared with and without the precipitation stage. DNA yield was measured as ng DNA recovered per mg wet gill, so as to ensure a fair comparison of DNA recovery across different gill weights.

### DNA extraction from gills and mucus samples

DNA was extracted from the gills at each of the seven timepoints (*n* = 105) according to the optimised protocol (each fish gill was processed separately and without regard to their gill score or the qPCR results). Mucus samples, including the possible replicates, from each of T2-T6 (*n* = 30) were processed using the extraction kit (AllPrep DNA and RNA extraction kit, QIAGEN, Hilden, Germany) according to the manufacturer’s instructions. Concentration of double-stranded DNA was determined using a Qubit dsDNA BR Assay Kit (ThermoFisher Scientific, Paisley, UK), following the instructions provided.

### 16S rRNA gene sequencing and *N. perurans* 18S rRNA gene quantification

The V4 region of 16S rRNA gene from gill and mucus DNA samples were amplified using the oligonucleotide primers 515F (5′-GTGCCAGCMGCCGCGGTAA-3′) and 806R (5′-GGACTACHVGGGTWTCTAAT-3′)^53^. PCR mixtures (25 µl final volume) contained: 12.5 ng genomic DNA, 0.2 µM (final concentration) of each primer (515F and 806R), and 12.5 µl 2X KAPA HiFi Hot Start Ready Mix (0.25 U; Roche). The PCR comprised of initial denaturation at 95 °C for 3 min; 25 cycles of 95 °C for 30 s, 55 °C for 30 s and 72 °C for 30 s; and a final incubation at 72 °C for 5 min. PCR products were purified (AMPure XP for PCR Purification, Beckman Coulter, USA) before adding Illumina sequencing adapters and indices using a Nextera XT Index Kit (Cambridge, United Kingdom) following the manufacturer’s instructions. A second PCR purification was carried out before quantifying the DNA using a 2100 Bioanalyzer system (Agilent Technologies), normalising the DNA concentration (to 4 nM) and pooling the libraries with unique indices. Pooled libraries were denatured (0.2 N NaOH), diluted and heat-denatured before sequencing. Amplicons were sequenced using an Illumina MiSeq platform^54^ using 5% PhiXas as internal control, following Illumina’s recommendations.

The concentration of *N. perurans* in gill and mucus samples was estimated in quantitative PCR assays targeting partial 18S rRNA gene sequences specific to the amoeba and using the primers NP1 (5′-AAAAGACCATGCGATTCGTAAAGT-3′) and NP2 (5′-CATTCTTTTCGGAGAGTGGAAATT-3′), with the NPP (6-FAM-ATCATGATTCACCATATGTT-MGB) probe, according to the procedure as described in detail by^13^. Each PCR mixture (25 μl) contained 5 µl microbial genomic DNA (5 ng/µl), 12.5 μl TaqMan® Universal 2 Master Mix (Applied Biosystems), and final concentrations of 300 nM primer NP1, 900 nM primer NP2 and 200 nM probe NPP. Each qPCR program comprised of an initial denaturation at 95 °C for 15 min, followed by 45 cycles of 95 °C for 15 s and 56 °C for 30 s in a real-time PCR thermocycler (Applied Biosystems AB7500). Positive and negative controls were included with each run, as internal and external process controls^13^.

It is worth mention that the present study considered as AGD-affected fish those salmon with a gill score of ≥ 1 and a positive qPCR result. Although a further confirmation using a histological approach would be desirable, previous studies have used only gill scoring and qPCR results to define AGD cases^34,35^.

### Bioinformatics and statistical analyses

Operational taxonomic units (OTUs) were constructed at 97% similarity using the VSEARCH workflow after pre-filtering stages including the removal of host contamination. In the final analysis, all clean OTUs were extracted for the samples that were not dropped because of their low number of reads (*n* = 129). Multivariate statistical analyses were performed on them using R in view of the metadata collected in this study. The details of the bioinformatics steps, along with procedures for the statistical analyses of fish features, qPCR data and environmental parameters, as well as software and R packages used, are provided in the Supplementary Methods (Supplementary File).

### Ethics declarations

Sampling of fish used for this study followed guidelines from the Health Products Regulatory Authority (www.hpra.ie) of Ireland based on legislation protecting animals used for scientific purposes. The sampling of tissues was conducted at the fish farm in question as part of routine farm practices and animal husbandry, and in line with approved methods of killing (i.e. anaesthetic overdose) and with proof of death (waiting for the onset of rigor mortis), according to Annex IV of the EU Directive Dir10/63/EU (as transposed into Irish legislation), with subsequent sectioning the relevant gill tissue. No procedure was conducted on live fish. This project took advantage of scheduled sampling, and no fish were sampled solely for the purpose of this project. Thus, no additional ethical review, or project authorisation, specifically for our work, was required from the regulatory body (the HPRA) or locally from the Animal Care Research Ethics Committee (ACREC) at NUI Galway. Equally, therefore, the use of the tissue was maximised, adhering to the three pillars (refine, reduce, replace) of animal welfare and research ethics.

### Consent for publication

All authors give their consent to publish the present research article.

## Supplementary Information

Supplementary Information 1.

Supplementary Information 2.

Supplementary Information 3.

## Data Availability

The sequencing data are available in the European Nucleotide Archive under the study accession number PRJEB32307 (http://www.ebi.ac.uk/ena/data/view/PRJEB32307), with further detail in the supplementary files.

## Abbreviations

AGD: Amoebic gill disease
CGD: Complex gill disease
DNA: Desoxiribonucleic acid
PCR: Polymerase chain reaction
qPCR: Quantitative polymerase chain reaction
EDTA: Ethylenediaminetetraacetic acid
OTU: Operational taxonomic unit
PERMANOVA: Permutational analysis of variance
ANOVA: Analysis of variance
