# Supplementary material for: Dynamic gill and mucus microbiomes during a gill disease episode in farmed Atlantic salmon
Source: Sci Rep. 2022 Oct 6;12:16719. doi: 10.1038/s41598-022-17008-2 (PMC9537138; doi:10.1038/s41598-022-17008-2)
Supplement: Supplementary file 3 — Supplementary Information 3. [file 41598_2022_17008_MOESM3_ESM.docx]

**DYNAMIC GILL AND MUCUS MICROBIOMES DURING A GILL DISEASE EPISODE IN FARMED ATLANTIC SALMON**

**Victor B. Birlanga^1^, Grace McCormack^2,3^, Umer Z. Ijaz^4^, Eugene MacCarthy^5^, Cindy Smith^4^, Gavin Collins^1,3^**

^1^Microbiology, School of Natural Sciences National University of Ireland Galway, University Road, Galway, H91 TK33, Ireland.

^2^Zoology, School of Natural Sciences National University of Ireland Galway, University Road, Galway, H91 TK33, Ireland.

^3^Ryan Institute, National University of Ireland Galway, University Road, Galway, H91 TK33, Ireland.

^4^Infrastructure and Environment, School of Engineering, University of Glasgow, Rankine Building, Oakfield Avenue, Glasgow, G12 8LT, United Kingdom

^5^Institute of Science, Technology and Medicine, Galway-Mayo Institute of Technology, Galway, H91 T8NW, Ireland

**Corresponding author:** Victor B. Birlanga

e-mail: v.birlanga1@nuigalway.ie

[tel: +353](tel:+353) 892 093 159

**Supplementary File**

**Supplementary Methods**

*Bioinformatics*

The software VSEARCH v2.3.4 (steps documented in <http://github.com/torognes/vsearch/wiki/VSEARCH-pipeline>) was used to generate the abundance table by constructing operational taxonomic units (OTUs), a proxy for species. Prior to using VSEARCH, the paired-end reads were preprocessed according to the recommendations given in author’s previous publications [1, 2] which results in significant reduction of substitution errors. Briefly, the paired-end reads were trimmed and filtered using Sickle v1.200 [3] by using a sliding window approach and trimming the reads where the average base quality drops below 20. Only the reads that were above 10 bp length were kept after trimming. Next, BayesHammer [4] was used from the Spades v2.5.0 assembler, which error-corrected the paired-end reads. Following this, pandaseqv(2.4) [5] was used to assemble the forward and reverse reads into a single sequence spanning the entire V4 region with a minimum overlap of 10 bp. The preprocessed reads (overlapped) from each sample were pooled together while barcodes were added to keep track of which sample the read originated from. The reads were then dereplicated, sorted in order of decreasing abundance and singletons were discarded. Next, the reads were clustered based on 97% similarity followed by a removal of clusters which had chimeric models built from more abundant reads (--uchime_denovo option in vsearch). To remove any chimeras that may have been missed, particularly in the case that they had parents that were absent from the reads or were present in very low abundance, a reference-based chimera filtering step (--uchime_ref option in vsearch) using a gold database (https://www.mothur.org/w/images/f/f1/Silva.gold.bacteria.zip) was applied. Finally, the OTU table was generated by matching the original barcoded reads against clean OTUs (a total of 17,428 OTUs for n=135 samples) at 97% similarity (a proxy for species-level separation). Having obtained the OTUs, we then used DeConseq [6] to identify OTUs that were contaminants, hitting on *Salmo salar* reference genome. This step identified 13,211 OTUs as contaminants bringing the total OTUs down to 4,217 OTUs. Then, the OTUs were manually checked against NCBI for further contaminants and filtered out some that were still hitting *Salmo salar* reference genome even after DeConseq and others to mitochondria leading to 3,852 OTUs For ensuing statistical analysis, any sample was dropped with total reads <2000 (6 samples in this dataset) and that resulted in a 129 (samples) X 3,834 (OTUs) abundance table.

The assign_taxonomy.py script from the Qiime workflow [7] was used to taxonomically classify the representative OTUs against the SILVA SSU Ref NR database release v123 database. After, the OTUs were multisequence aligned using MAFFT v 7.3 [8] and were used in FastTree v2.1.7 [9] to generate the phylogenetic tree in NEWICK format. The biom file for the OTUs was then generated by combining the abundance table with taxonomy information using make_otu_table.py from the Qiime workflow.

*Statistical Analyses*

Statistical analyses were performed in R using the combined data generated from the bioinformatics as well as meta data associated with the study (environmental factors, fish features, and qPCR results). The vegan package [10] was used for alpha and beta diversity analyses. For alpha diversity measures we have used: Shannon entropy – a commonly used index to measure balance within a community, and rarefied richness (exponential of Shannon entropy) – the estimated number of species, and both indices after rarefying the abundance table to minimum library size. Ordination of OTU table in reduced space (beta diversity) was done using Principal Coordinate Analysis (PCoA) plots of OTUs. Three different distance measures were made using Vegan’s cmdscale() function: (1) Bray-Curtis is a distance metric which considers only OTU abundance counts, (2) Unweighted Unifrac is a phylogenetic distance metric which calculates the distance between samples by taking the proportion of the sum of unshared branch lengths in the sum of all the branch lengths of the phylogenetic tree for the OTUs observed in two samples, and without taking into account their abundances and, (3) Weighted Unifrac is a phylogenetic distance metric combining phylogenetic distance with relative abundances. This places emphasis on dominant OTUs or taxa. Unifrac distances were calculated using the phyloseq package [11].

Analysis of variance for explanatory variables (or sources of variation) was performed using Vegan’s adonis() against distance matrices (Bray-Curtis/UnweightedUniFrac/Weighted UniFrac). This function, referred to as PERMANOVA, fit linear models to distance matrices and used a permutation test with pseudo-F ratios. To give an account of environmental filtering (phylogenetic overdispersion versus clustering), phylogenetic distances within each sample were further characterised by calculating the nearest taxa index (NTI) and net relatedness index (NRI). This analysis helped determine whether the community structure was stochastic (overdispersion and driven by competition among taxa) or deterministic (clustering and driven by strong environmental pressure). The NTI was calculated using mntd() and ses.mntd(), and the mean phylogenetic diversity (MPD) and NRI were calculated using mpd() and ses.mpd() functions from the picante package [12]. NTI and NRI represent the negatives of the output from ses.mntd() and ses.mpd(), respectively. Additionally, they quantify the number of standard deviations that separate the observed values from the mean of the null distribution (999 randomisation using null.model-‘richness’ in the ses.mntd() and ses.mpd() functions and only considering taxa as either present or absent regardless of their relative abundance). Based upon the recommendations given [12], only the top 1000 most abundant OTUs were used for the calculations.

Discriminant analyses were performed for two cases: longitudinal comparison of gill microbiome, and cross-sectional comparison of microbiome between Gill and Mucus samples. For the first case, Sparse Projection to Latent Structure – Discriminant Analysis (sPLS-DA) was used with the R’s mixOmics package [14] The procedure constructs artificial latent components of the predicted dataset (genera table denoted as $X\left( N\times P \right)$collated at genus level) and the response variable (denoted as $Y$with categorical information of samples, e.g. T0, T1, T2, T3, T4, T5, and T6 for longitudinal analysis) by factorizing these matrices into scores and loading vectors in a new space such that the covariance between the scores of these two matrices$cov\left( X_{h}a_{h},Y_{h}b_{h} \right)$ in this space is maximized under two constraints: $\left\| a_{h} \right\|_{2}=1$; and $\left\| a_{h} \right\|_{1}\leq\lambda$, where $a_{h}$ and $b_{h}$are the corresponding loading vectors for $X$ and $Y$, and $h$ represents the number of components (akin to PCA analysis). The first constraint ensures the loading vector to have unit magnitude (requirement of the procedure) and the second constraint (also called $l_{1}$ penalty) to ensure that for the features that do not vary between the categories, the corresponding loading vector coefficients go to zero. This is done by using the sparsity control parameter $\lambda$ in the above equation, and by adjusting it enforces shrinkage of loading vector coefficients. According to the recommendations given in mixOmics package (http://www.mixomics.org), before applying the procedure splsda(), we pre-filter 1% of the lowest abundant genera and then perform TSS+CLR (Total Sum Scaling followed by Centralised Log Ratio) normalisation. To predict the number of latent components (associated loading vectors) and the number of discriminants, the perf.plsda() and tune.splsda() functions were used, respectively. In the latter case, we fine tune the model was applied using leave-one-out cross-validation by splitting the data into training and testing sets and then finding the classification error rates employing two metrics, overall error rates and balanced error rates (BER), between the predicted latent variables with the centroid of the class labels (categories considered in this study) using the centroid distance. BER accounts for differences in number of samples between different categories.

For the second case (Gill vs Mucus), the Multivariate Integration (MINT) algorithm [15] was used, which is an extension of the multi-group Projection to Latent Structure (mgPLS), and it attempts to find a common projection space across all studies, defined on a small subset of discriminative variables that consistently discriminate the outcome classes (timepoints T2 to T6). In MINT, we have combined $M=2$ datasets denoted $X^{\left( 1 \right)}\left( N_{1}\times P \right)$, $X^{\left( 2 \right)}\left( N_{2}\times P \right)$ for Gill and Mucus, respectively, where both the datasets share the P OTUs whilst the number of samples differ, i.e., $N_{1}$, $N_{2}$. Both studies have associated dummy indicator outcome $Y^{\left( 1 \right)}$, $Y^{\left( 2 \right)}$ in which all the timepoints (T2, T3, T4, T5, and T6 only as T0, and T1 were not available for Mucus samples) are represented. MINT then solves the problem: $\begin{aligned} \text{max} \\ a_{h},b_{h} \end{aligned}\sum_{m=1}^{M} N_{m}\text{cov}\left( X_{h}^{\left( m \right)}a_{h},Y_{h}^{\left( m \right)}b_{h} \right)$, with the previous constrains$\left\| a_{h} \right\|_{2}=1$ and $\left\| a_{h} \right\|_{1}\leq\lambda$, where the covariance of scores between the datasets are maximised by finding the global loading vectors $a_{h}$ and $b_{h}$ common to all studies. The prefiltering and the cross-validation procedure is similar to the previous case (sPLS-DA).

The “BVSTEP” routine [16] was used to search for the highest correlation, in a Mantel test, between dissimilarities of fixed and multivariate datasets. To run this algorithm, bvStep() (from the sinkr package) [17] was used. It permuted through 2n-1 possible combinations of features in the variable dataset. Rather than testing all possible OTU subsets (for n=3,834) which is intractable, the top 1000 most abundant OTUs [18] were used to best correlate dissimilarities (Bray-Curtis distance) of samples using subsets against dissimilarities of the samples given to all the OTUs. This analysis is complementary to the sPLS-DA and MINT which identified the OTUs causing major shifts in beta diversity but without considering their labelling (i.e., timepoints) with the intention to identify OTUs that change markedly in the sample space.

Subset regression analysis of the microbial community was also performed using the leaps package [19], which performs exhaustive search for the best subsets of the explanatory variables (meta data recorded in this study) for predicting the dependent variable in linear regression using an efficient branch-and-bound algorithm. For cross-validation, CVlm() from R’s DAAG package was used [20]. Other than TSS+CLR normalisation for the abundance table, Log10 normalisation for qPCR data was used, and Arcsinh normalisation for the cell count data as is the general practice.

In majority of the figures displaying boxplots, pair-wise ANOVA was performed taking two categories at a time, and where significant (p ≤ 0.05), joined them together by a line and plotting significance on top (*: 0.01 ≤ p < 0.05; **: 0.001 ≤ p < 0.01; ***: p < 0.001).

**Supplementary Figures and Tables**


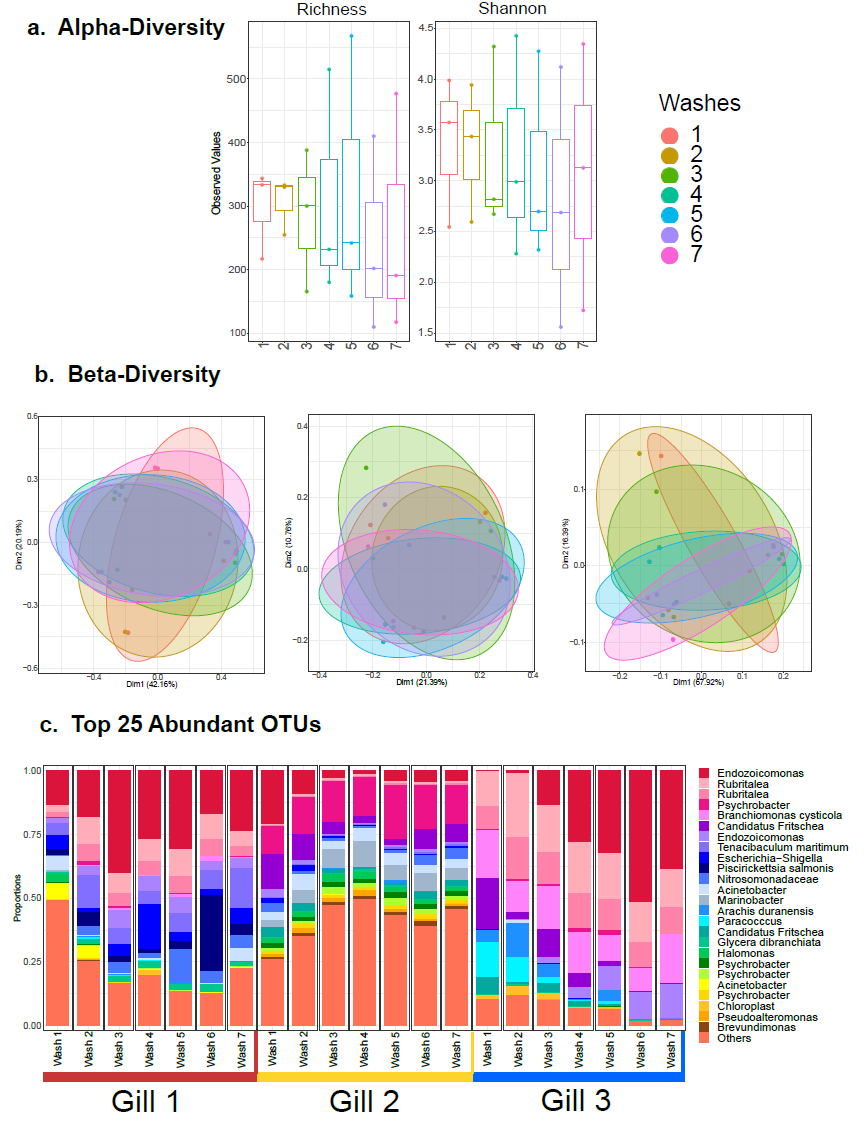


**Supplementary Figure 1**: **Microbial diversity and community structure for the First step of the Nucleic acid extraction protocol optimisation**. **(a)** and **(b)** represent alpha diversity and beta diversity indices, respectively. In (b), the ellipses are drawn at 95% confidence interval of standard error with lines going from Wash number 1 to Wash number 7 plotted at mean of the ordination values at each wash. From the left graphic until the right one: Bray-Curtis; UniFrac; Weighted UniFrac. **(c)** shows bacterial community structure based on relative abundance of the top-25 most abundant OTUs from across each Wash in three gill tissue samples, where ‘others’ refers to all OTUs not included in the ‘top-25’; no significant difference where found in the alpha diversity analyses between washes. This figure was created using Inkscape, version 0.92.4 (https://inkscape.org/).


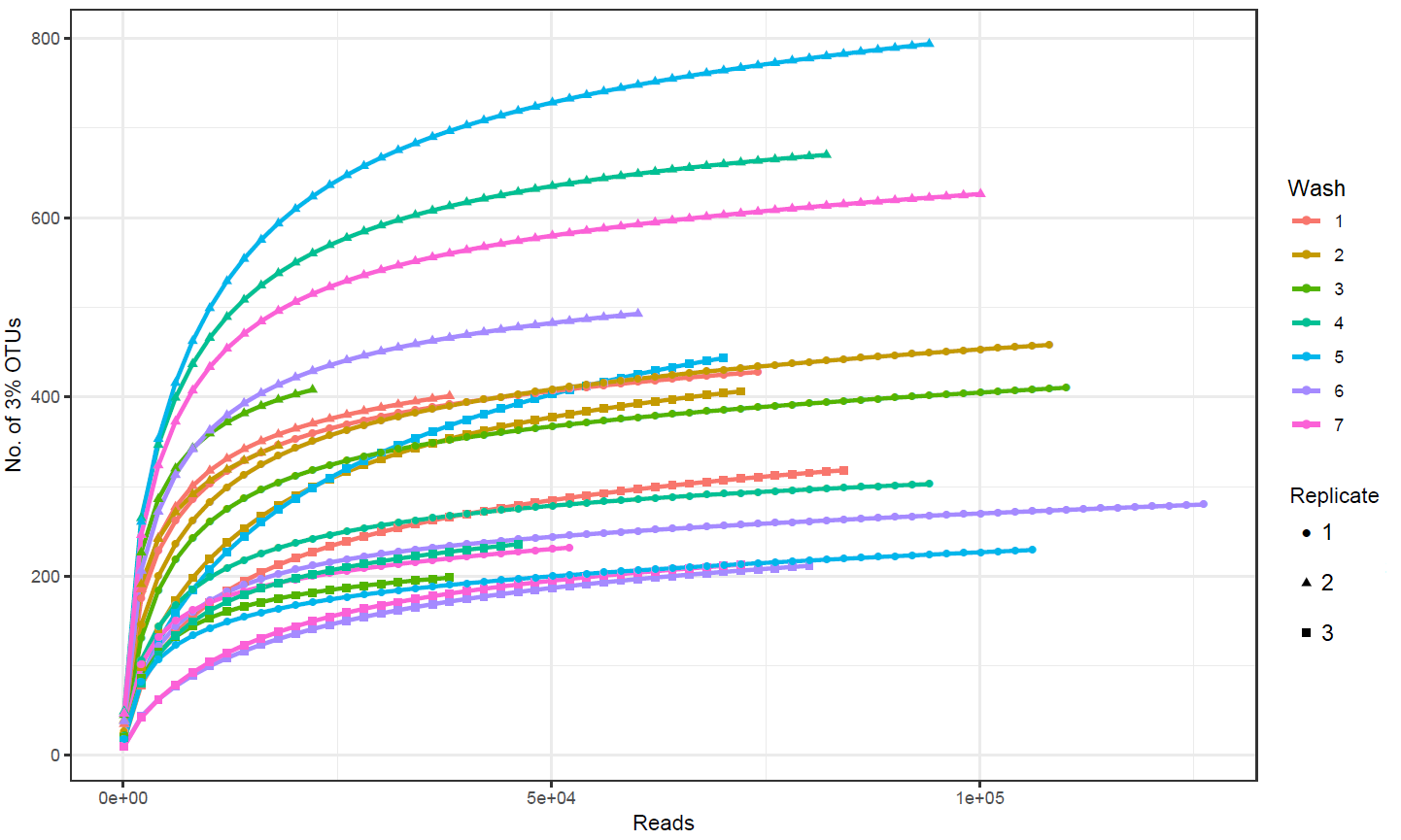


**Supplementary Figure 2:** Rarefaction curves for 3% OTUs. These curves were generated by using rarefy() function from R’s vegan package that gives the expected species richness in random subsamples of OTU table. This figure was created using Inkscape, version 0.92.4 (https://inkscape.org/).


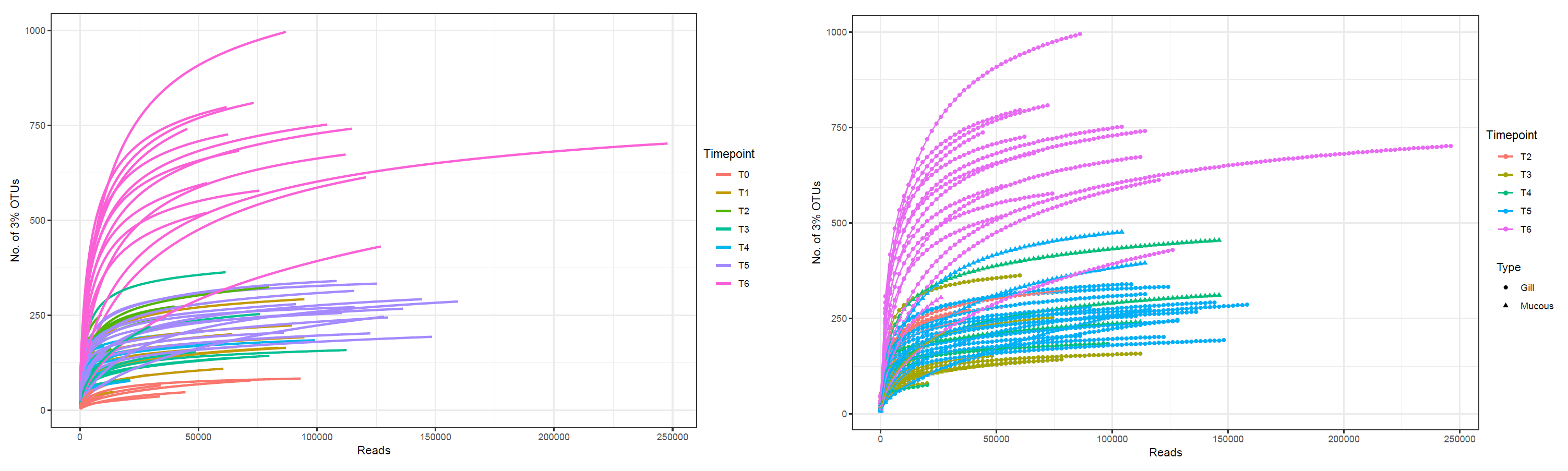
**Supplementary Figure 3:** Rarefaction curves for 3% OTUs. Details are given in legend of Supplementary Figure 2. This figure was created using Inkscape, version 0.92.4 (https://inkscape.org/).

**
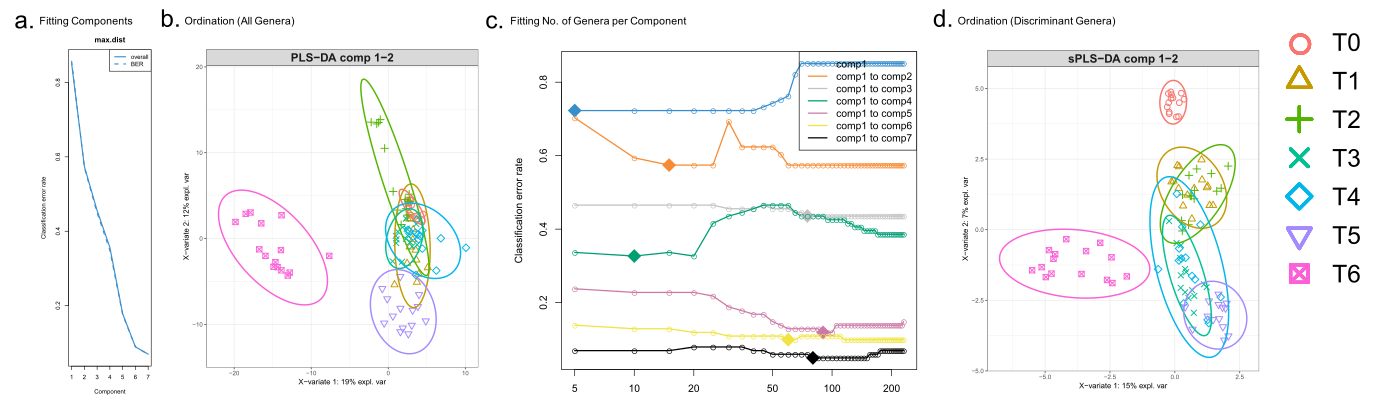

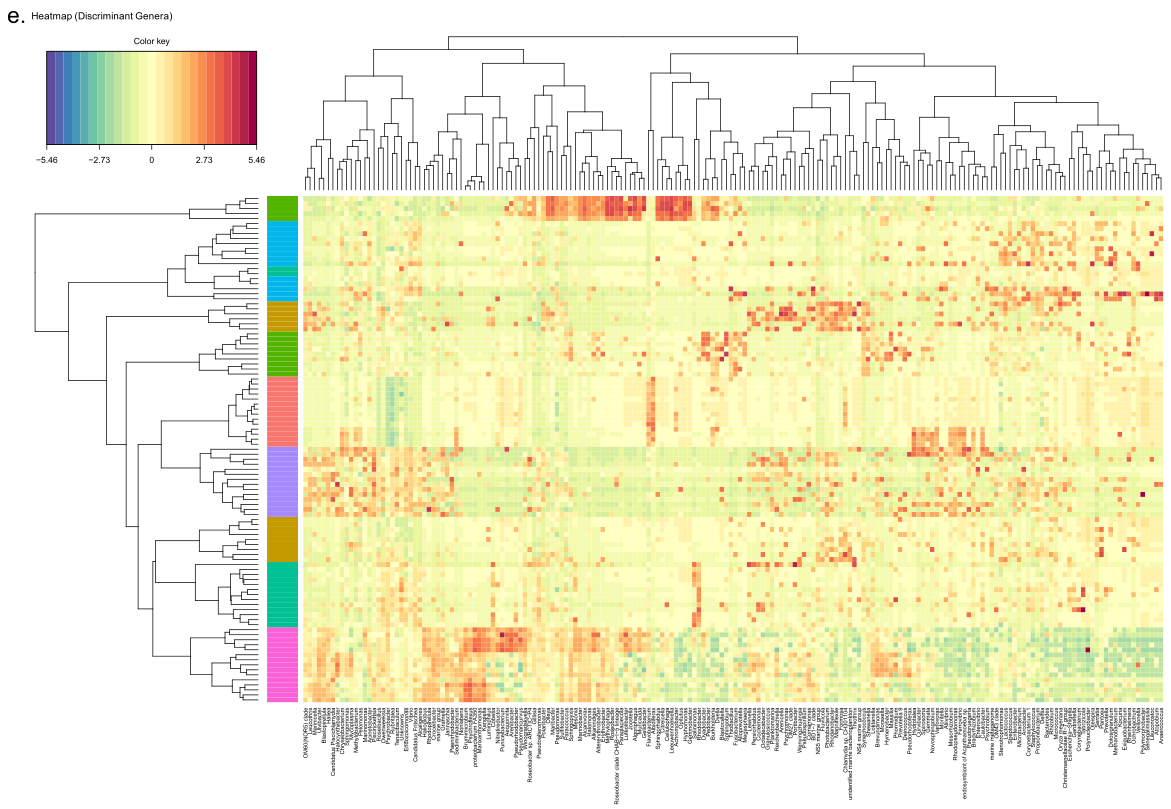
Supplementary Figure 4**: **sPLS-DA Gill Longitudinal Data Analysis**. The algorithm is a two-step process where **(a)** seven components were found reducing the classification error rates (using max.dist in the function) in the algorithm, with **(b)** showing the ordination of samples using all the genera in the first two components (PLS-DA) with ellipses representing 95% confidence interval and percentage variations explained by these components in axes labels. In step two, **(c)** the number of discriminating genera were found for each component, highlighted as diamonds. **(d)** is similar to **(b)** however the ordination was considered using the discriminants from all seven components (sPLS-DA); **(e)** shows the heatmaps of these discriminant genera, with both rows and columns ordered using hierarchical (average linkage) clustering to identify blocks of genera of interest. Heatmap depicts TSS+CLR normalised abundances: high abundance (red) and low abundance (blue). This figure was created using Inkscape, version 0.92.4 (<https://inkscape.org/>). A high-resolution file with this figure in is available.

**
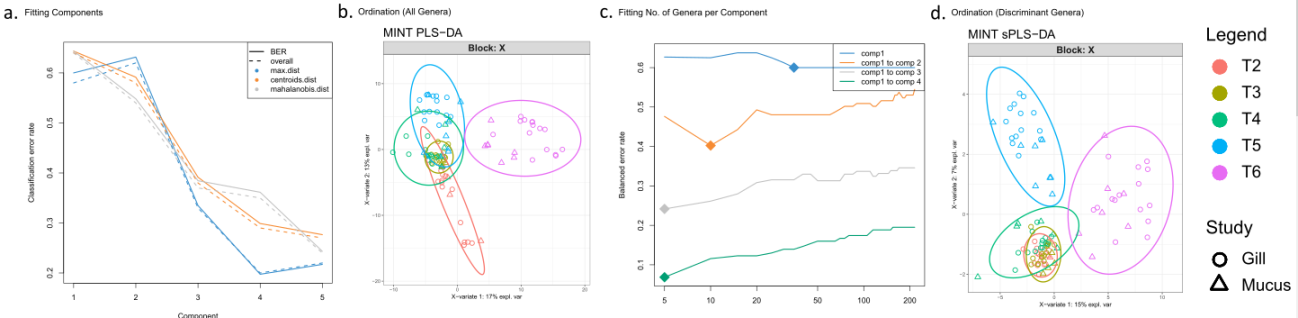

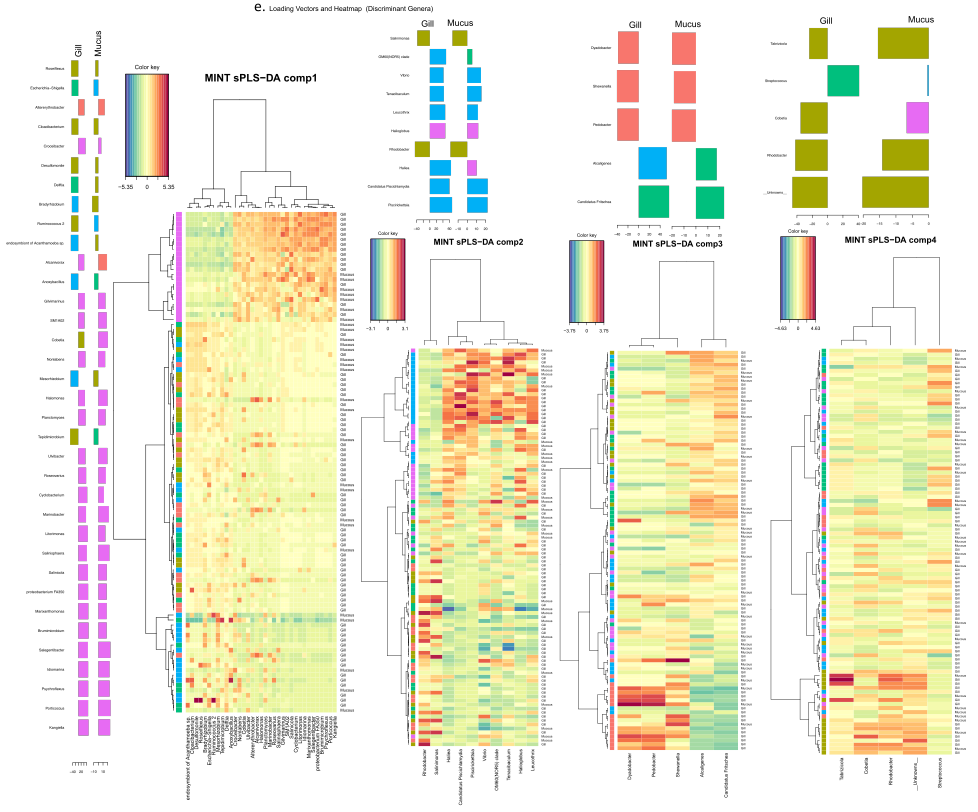
Supplementary Figure 5**: **MINT study-wise discriminant analysis (Gill vs Mucus)**. The algorithm is a two-step process where **(a)** four components were found that reduce the classification error rates (using max.dist in the function) in the algorithm, with **(b)** showing the ordination of samples using all the genera in the first two components (MINT PLS-DA) with ellipse representing 95% confidence interval and percentage variations explained by these components in axes labels. In step two, **(c)** the number of discriminating genera were found for each component, highlighted as diamonds. **(d)** is similar to **(b)** however the ordination was considered using the discriminants from all four components (MINT sPLS-DA); **(e)** shows the MINT sPLS loading vectors $a_{1}$,$a_{2}$,$a_{3}$, and $a_{4}$with non-zero weights for component 1, 2, 3, and 4 for both Gill and Mucus together, along with the heatmaps of these discriminant genera, with both rows and columns ordered using hierarchical (average linkage) clustering to identify blocks of genera of interest. Heatmap depicts TSS+CLR normalised abundances: high abundance (red) and low abundance (blue). Loading vectors are coloured by time points with maximal abundance. This figure was created using Inkscape, version 0.92.4 (<https://inkscape.org/>). A high-resolution file with this figure in is available.

**Supplementary Table 1: Most Abundant and Relevant OTUs (Operational Taxonomical Units) Description.** This table only show the top 25 most abundant OTUs/Genus (according to **Fig. 4** in the main manuscript) and the most relevant taxa that appear to consistently being selected in subsets of species explaining the variability in sample space (beta diversity), as well as their OTU identification numbers. The time when the OTU was more abundant is showed in the “Significance” column. The type of sample where the OTU abundance was higher is showed in the “Type of sample” column.

| **OTU ID** | **OTU/Genera** | **Significance** | **Type of sample** | **Description** |
| --- | --- | --- | --- | --- |
| 3352 | Rubritalea | Present during the whole period, decrease in T4 | Gill & Mucus | Was found on salmon, seabram and seabass gills, skin and guts [21, 22, 23], even on some salmon affected by epitheliocystis [21] |
| 4 | Albidiferax | Abundance increases during the gill disease, but seems like freshwater bath treatments reduce its abundance | More abundant in Mucus | This genus appeared very commonly on rainbow trout eggs [24], and associated with biofilms [25] |
| 11 | Endozoicomonas | Abundance increases during the gill disease | More abundant on Gills | First described as a causative agent for epitheliocystis [26], even on Atlantic salmon gills [27, 28] |
| 24 | Nitrosomonas | Abundance increases after the the gill disease, maximum at T3 | Gill & Mucus | Was found more often in water samples [29]. But this genus was detected on Atlantic salmon gills in a Epitheliocystis outbreak [30] |
| 9 | C. Branchiomonas cysticola | Only found during the the gill disease | Gill & Mucus | Very common agent of Epitheliocystis on Atlantic salmon gills [31]. This genus was found associated with “Proliferative Gill Inflammation” (PGI) and “Complex Gill Disease” (CGD) [32] |
| 10 | Psychrobacter | Appeared before the gill disease, and abundance increases during the gill disease | Gill & Mucus | This genus was found on affected and non-affected AGD Atlantic salmon gills [27], gut [33], skin [34], and even kidney [35] |
| 7011 | Tenacibaculum maritimum | Mainly present during the gill disease | Gill & Mucus | Is the causative agent of tenacibaculisis, especially at low water temperatures [32]. Was found on skin, gill and salmon fins [36], even in freshwater [34]. Another species from the same genus was associated with AGD in a laboratory trial [72] |
| 29 | Vibrio | Mostly present during the gill disease | Gill & Mucus | *Vibrio* sp. was found to appear on Atlantic salmon epidermal mucus [37] and gastrointestinal microbiome [38]. Also closely associated with *N. perurans* in *in vitro* cultures [71] |
| 20 | Piscirickettsia salmonis | Mainly present during the gill disease | Gill & Mucus | It causes Piscirickettsiosis in Atlantic salmon [39], common to find on gills [40] |
| 15 | Flavobacterium | Only appeared during the gill disease, and abundance increases in T4 and T5 | Gill & Mucus | This genus appeared before on Atlantic salmon gills [27], gut [22], and skin [34] |
| 22 | Pseudomonas | Present during the whole period | Gill & Mucus | Was present in Atlantic salmon gut [22], gills [27], and even in freshwater salmon skin [34] |
| 17 | C. Fritschea | Present during the whole period in low abundance, but abundance increases at T2 | Gill & Mucus | Was found to be very close to other gill pathogens in Atlantic salmon [41], such as *Candidatus* Syngnamydia *venezia* and *Candidatus Syngnamydia salmonis* [42, 43], associated with *Paramoeba perurans* |
| 23 | Olleya | Abundance increases at T2 in one of the cages | Gill & Mucus | Typically present in seawater [44], was not related with any Atlantic salmon microbiome before |
| 111 | Staphylococcus | Present during the whole period in low abundance, but abundance increases at T2 | Gill & Mucus | This genus was found on Atlantic salmon skin [34] and AGD affected gills [27] |
| 12 | Alcanivorax | Present during the whole period in low abundance, but abundance increases at T4 | More abundant on Gills | Was found in gut microbiome of *Epinephelus coioides* before and after a probiotic treatment with *Psychrobacter* [45]. Was detected in marine sediments [46] |
| 477 | Winogradskyella | In low abundance during the gill disease, maximum at T6 | Gill & Mucus | This genus was suggested to enhance the severity of the AGD [47], but a further study discarded the idea of that connection [48] |
| 105 | Shewanella | Present during the whole period in low abundance, but abundance increases at T2 and T6 | Gill & Mucus | This genus was found in Atlantic cod intestine [49], and in other fish skin and gill microbiome studies [50, 51] |
| 1011 | Delftia | Present in low abundance during the gill disease, maximum at T6 | Gill & Mucus | Was found in the Atlantic salmon intestine, and skin microbiome [52] |
| 35 | Pelomonas | Present in low abundance during the gill disease, maximum at T6 | Gill & Mucus | This genus is part of the proposed core microbiome in intestine of fish [53, 54], being present in the *Gambusia affinis* skin microbiome [55] |
| 1970 | Pedobacter | Present in low abundance during the gill disease, maximum at T2 | Gill & Mucus | This genus appears in the gut microbiome of some tropical fish [54] with *Bacillus* and *Vibrio* species too |
| 72 | Synechococcus | Present in low abundance in the gill disease, maximum at T6 | Gill & Mucus | This genus was found in Atlantic mackerel gills [50], and in Chinook salmon gut microbiome [56] |
| 54 | Marinobacter | Present during the whole period | Gill & Mucus | This genus was detected in AGD affected and non-affected Atlantic salmon gills [27]. In another study [57], was associated with *Paramoeba perurans* cultures |
| 268 | Lactobacillus | Significant due to being selected in subsets of species explaining the variability in beta diversity | Gill & Mucus | Is a genus very commonly present with high abundance in skin [53] and gut [58] microbiome of different fish |
| 145 | Turicibacter | Significant due to being selected in subsets of species explaining the variability in beta diversity | Gill & Mucus | Was found in the intestine of different fish [59, 60] |
| 62 | Stenotrophomonas | Significant due to being selected in subsets of species explaining the variability in beta diversity | Gill & Mucus | Can be found in Atlantic salmon skin [34] and intestine [61] microbiome |
| 136 | Bifidobacterium | Significant due to being selected in subsets of species explaining the variability in beta diversity | Gill & Mucus | This bacteria was not related with fish microbiome before, but is commonly used as a potential probiotic for fish [62] |
| 347 | Allobaculum | Significant due to being selected in subsets of species explaining the variability in beta diversity | Gill & Mucus | Was detected in sediment and water samples in China [63], but could not be found in any fin-fish microbiome study |
| 248 | Propionibacterium | Significant due to being selected in subsets of species explaining the variability in beta diversity | Gill & Mucus | Was found before on AGD affected and non-affected Atlantic salmon gills [27]. This genus was detected widely across mucus and tissue samples of Atlantic salmon, seabass, and seabream gut [23, 61] |
| 188 | Gardnerella | Significant due to being selected in subsets of species explaining the variability in beta diversity | Gill & Mucus | Could not be found in any fish microbiome study |
| 3156 | Illumatobacter | Significant due to being selected in subsets of species explaining the variability in beta diversity | Gill & Mucus | This uncultured actinobacteria was found in coastal and oceanic sediments [64], and in a wide range of marine organisms [65] |
| 305 | Altererythrobacter | Significant due to being selected in subsets of species explaining the variability in beta diversity | Gill & Mucus | This genus was found in Atlantic salmon gut microbiome after a fermented soybean meal diet [66] |
| 1802 | Dyadobacter | Significant due to being selected in subsets of species explaining the variability in beta diversity | Gill & Mucus | This genus was found on the surface of Lake Sturgeon eggs after an iodine disinfection treatment [67] |
| 1344 | Rhodobacter | Significant of being  in subsets of species explaining the variability in beta diversity | Gill & Mucus | Are abundant in fresh and seawater samples and in intestine, skin, and gills from Atlantic salmon [22, 27, 34] |
| 3149 | Psychroserpens | Significant due to being selected in subsets of species explaining the variability in beta diversity | Gill & Mucus | Was mainly found in AGD affected Atlantic salmon gills [27], and it was proposed to be an opportunistic pathogen associated with AGD. |
| 522 | Salinimonas | Significant due to being selected in subsets of species explaining the variability in beta diversity | Gill & Mucus | Was previously found in marine sediments [68], but could not be found in any fish microbiome study |
| 754 | Burkholderia | Significant due to being selected in subsets of species explaining the variability in beta diversity | Gill & Mucus | This genus was found in healthy gut [22, 61], skin [34], and on gills during a “Proliferative gill inflammation” [28] from Atlantic salmon |
| 807 | Dyella | Significant due to being selected in subsets of species explaining the variability in beta diversity | Gill & Mucus | This bacteria was found in the digestive cavity of the jellyfish *Cotylorhiza tuberculata* [69], but could not be found in any fin-fish microbiome study |
| 73 | Piscichlamydia | Significant due to being selected in subsets of species explaining the variability in beta diversity | Gill & Mucus | This genus was found to have a positive association with the “Proliferative Gill Inflammation” [28] on Atlantic salmon gills. Was also related with Epitheliocystis [70] |

**References**

1. Schirmer M, Ijaz UZ, D'Amore R, Hall N, Sloan WT, Quince C. Insight into biases and sequencing errors for amplicon sequencing with the Illumina MiSeq platform. Nucleic acids research. 2015;43(6):e37-.
2. D’Amore R, Ijaz UZ, Schirmer M, Kenny JG, Gregory R, Darby AC, Shakya M, Podar M, Quince C, Hall N. A comprehensive benchmarking study of protocols and sequencing platforms for 16S rRNA community profiling. BMC genomics. 2016;17(1):55.
3. Joshi NA, Fass JN. Sickle: A sliding-window, adaptive, quality-based trimming tool for FastQ files (Version 1.33)[Software]. 2011.
4. Nikolenko SI, Korobeynikov AI, Alekseyev MA. BayesHammer: Bayesian clustering for error correction in single-cell sequencing. InBMC genomics. BioMed Central. 2013;14(1):7.
5. Masella AP, Bartram AK, Truszkowski JM, Brown DG, Neufeld JD. PANDAseq: paired-end assembler for illumina sequences. BMC bioinformatics. 2012;13(1):31.
6. Schmieder R, Edwards R. Fast identification and removal of sequence contamination from genomic and metagenomic datasets. PloS one. 2011;6(3).
7. Caporaso JG, Kuczynski J, Stombaugh J, Bittinger K, Bushman FD, Costello EK, Fierer N, Pena AG, Goodrich JK, Gordon JI, Huttley GA. QIIME allows analysis of high-throughput community sequencing data. Nature methods. 2010;7(5):335.
8. Katoh K, Standley DM. MAFFT multiple sequence alignment software version 7: improvements in performance and usability. Molecular biology and evolution. 2013;30(4):772-80.
9. Price MN, Dehal PS, Arkin AP. FastTree 2–approximately maximum-likelihood trees for large alignments. PloS one. 2010;5(3).
10. Oksanen J, Kindt R, Legendre P, O’Hara B, Stevens MH, Oksanen MJ, Suggests MA. The vegan package. Community ecology package. 2007;10:631-7.
11. McMurdie PJ, Holmes S. phyloseq: an R package for reproducible interactive analysis and graphics of microbiome census data. PloS one. 2013;8(4).
12. Kembel SW, Cowan PD, Helmus MR, Cornwell WK, Morlon H, Ackerly DD, Blomberg SP, Webb CO. Picante: R tools for integrating phylogenies and ecology. Bioinformatics. 2010;26(11):1463-4.
13. Stegen JC, Lin X, Konopka AE, Fredrickson JK. Stochastic and deterministic assembly processes in subsurface microbial communities. The ISME journal. 2012;6(9):1653-64.
14. Rohart F, Gautier B, Singh A, Lê Cao KA. mixOmics: An R package for ‘omics feature selection and multiple data integration. PLoS computational biology. 2017;13(11):e1005752.
15. Rohart F, Eslami A, Matigian N, Bougeard S, Le Cao KA. MINT: a multivariate integrative method to identify reproducible molecular signatures across independent experiments and platforms. BMC bioinformatics. 2017;18(1):128.
16. Clarke KR, Ainsworth M. A method of linking multivariate community structure to environmental variables. Marine Ecology-Progress Series. 1993;92:205-.
17. Taylor M. sinkr: Collection of functions with emphasis in multivariate data analysis. R package version. 2016;1(1).
18. Ijaz UZ, Sivaloganathan L, McKenna A, Richmond A, Kelly C, Linton M, Stratakos AC, Lavery U, Elmi A, Wren BW, Dorrell N. Comprehensive longitudinal microbiome analysis of the chicken cecum reveals a shift from competitive to environmental drivers and a window of opportunity for Campylobacter. Frontiers in microbiology. 2018;9:2452.
19. Lumley T, Miller A. Leaps: regression subset selection. R package version. 2009;2:2366.
20. Maindonald J, Braun WJ. DAAG: Data analysis and graphics data and functions. R Package Version 1.20. 2014.
21. Soto MG, Vidondo B, Vaughan L, Seth-Smith HM, Nufer L, Segner H, Rubin JF, Schmidt-Posthaus H. The emergence of epitheliocystis in the upper Rhone region: evidence for Chlamydiae in wild and farmed salmonid populations. Archives of microbiology. 2016;198(4):315-24.
22. Wang C, Sun G, Li S, Li X, Liu Y. Intestinal microbiota of healthy and unhealthy Atlantic salmon *Salmo salar* L. in a recirculating aquaculture system. Journal of Oceanology and Limnology. 2018;36(2):414-26.
23. Rosado D, Perez-Losada M, Severino R, Cable J, Xavier R. Characterization of the skin and gill microbiomes of the farmed seabass (*Dicentrarchus labrax*) and seabream (*Sparus aurata*). Aquaculture. 2019;500:57-64.
24. Heikkinen J, Tiirola M, Mustonen SM, Eskelinen P, Navia‐Paldanius D, von Wright A. Suppression of *Saprolegnia* infections in rainbow trout (*Oncorhynchus mykiss*) eggs using protective bacteria and ultraviolet irradiation of the hatchery water. Aquaculture research. 2016;47(3):925-39.
25. Lyons PP, Turnbull JF, Dawson KA, Crumlish M. Phylogenetic and functional characterization of the distal intestinal microbiome of rainbow trout *Oncorhynchus mykiss* from both farm and aquarium settings. Journal of Applied Microbiology. 2017;122(2):347-63.
26. Mendoza M, Güiza L, Martinez X, Caraballo X, Rojas J, Aranguren LF, Salazar M. A novel agent (*Endozoicomonas elysicola*) responsible for epitheliocystis in cobia *Rachycentrum canadum* larvae. Diseases of aquatic organisms. 2013;106(1):31-7.
27. Bowman JP, Nowak B. Salmonid gill bacteria and their relationship to amoebic gill disease. Journal of Fish Diseases. 2004;27(8):483-92.
28. Steinum T, Sjåstad K, Falk K, Kvellestad A, Colquhoun DJ. An RT PCR-DGGE survey of gill-associated bacteria in Norwegian seawater-reared Atlantic salmon suffering proliferative gill inflammation. Aquaculture. 2009;293(3-4):172-9.
29. Elizondo-Patrone C, Hernández K, Yannicelli B, Olsen LM, Molina V. The response of nitrifying microbial assemblages to ammonium (NH4+) enrichment from salmon farm activities in a northern Chilean Fjord. Estuarine, Coastal and shelf science. 2015;166:131-42.
30. Wiik-Nielsen J, Solheim HT, Steinum TM, Bornø G, Skjelstad HR, Olsen AB, Colquhoun DJ. A novel epitheliocystis associated bacterium in Atlantic salmon.
31. Mitchell SO, Steinum TM, Toenshoff ER, Kvellestad A, Falk K, Horn M, Colquhoun DJ. ‘*Candidatus* Branchiomonas cysticola’ is a common agent of epitheliocysts in seawater-farmed Atlantic salmon *Salmo salar* in Norway and Ireland. Diseases of aquatic organisms. 2013;103(1):35-43.
32. Herrero A, Thompson KD, Ashby A, Rodger HD, Dagleish MP. Complex gill disease: an emerging syndrome in farmed Atlantic salmon (*Salmo salar* L.). Journal of comparative pathology. 2018;163:23-8.
33. Askarian F, Zhou Z, Olsen RE, Sperstad S, Ringø E. Culturable autochthonous gut bacteria in Atlantic salmon (*Salmo salar* L.) fed diets with or without chitin. Characterization by 16S rRNA gene sequencing, ability to produce enzymes and in vitro growth inhibition of four fish pathogens. Aquaculture. 2012;326:1-8.
34. Lokesh J, Kiron V. Transition from freshwater to seawater reshapes the skin-associated microbiota of Atlantic salmon. Scientific reports. 2016;6:19707.
35. McCarthy Ú, Stagg H, Donald K, Garden A, Weir SJ. *Psychrobacter* sp. isolated from the kidney of salmonids at a number of aquaculture sites in Scotland. Bull Eur Assoc Fish Pathol. 2013;33:67-72.
36. Downes JK, Collins EM, Morrissey T, Hickey C, O’Connor I, Rodger HD, MacCarthy E, Palmer R, Ruttledge M, Ruane NM. Confirmation of *Neoparamoeba perurans* on the gills of Atlantic salmon during the earliest outbreaks of amoebic gill disease in Ireland. Bull. Eur. Assoc. Fish Pathol.. 2018;38(1).
37. Llewellyn MS, Leadbeater S, Garcia C, Sylvain FE, Custodio M, Ang KP, Powell F, Carvalho GR, Creer S, Elliot J, Derome N. Parasitism perturbs the mucosal microbiome of Atlantic Salmon. Scientific reports. 2017;7:43465.
38. Zarkasi KZ, Abell GC, Taylor RS, Neuman C, Hatje E, Tamplin ML, Katouli M, Bowman JP. Pyrosequencing‐based characterization of gastrointestinal bacteria of Atlantic salmon (*Salmo salar* L.) within a commercial mariculture system. Journal of applied microbiology. 2014;117(1):18-27.
39. Olsen AB, Melby HP, Speilberg L, Evensen Ø, Håstein T. Piscirickettsia salmonis infection in Atlantic salmon *Salmo salar* in Norway--epidemiological, pathological and microbiological findings. Diseases of aquatic organisms. 1997;31(1):35-48.
40. Bustos PA, Young ND, Rozas MA, Bohle HM, Ildefonso RS, Morrison RN, Nowak BF. Amoebic gill disease (AGD) in Atlantic salmon (*Salmo salar*) farmed in Chile. Aquaculture. 2011;310(3-4):281-8.
41. Pawlikowska-Warych M, Deptuła W. Characteristics of chlamydia-like organisms pathogenic to fish. Journal of applied genetics. 2016;57(1):135-41.
42. Nylund S, Steigen A, Karlsbakk E, Plarre H, Andersen L, Karlsen M, Watanabe K, Nylund A. Characterization of ‘*Candidatus* Syngnamydia salmonis’ (Chlamydiales, Simkaniaceae), a bacterium associated with epitheliocystis in Atlantic salmon (*Salmo salar* L.). Archives of microbiology. 2015;197(1):17-25.
43. Nylund A, Pistone D, Trösse C, Blindheim S, Andersen L, Plarre H. Genotyping of Candidatus Syngnamydia salmonis (chlamydiales; Simkaniaceae) co-cultured in *Paramoeba perurans* (amoebozoa; Paramoebidae). Archives of microbiology. 2018;200(6):859-67.
44. Nichols CM, Bowman JP, Guezennec J. *Olleya marilimosa* gen. nov., sp. nov., an exopolysaccharide-producing marine bacterium from the family Flavobacteriaceae, isolated from the Southern Ocean. International journal of systematic and evolutionary microbiology. 2005;55(4):1557-61.
45. Yang HL, Sun YZ, Ma RL, Li JS, Huang KP. Probiotic *Psychrobacter* sp. improved the autochthonous microbial diversity along the gastrointestinal tract of grouper *Epinephelus coioides*. J Aquac Res Development S. 2011;1:001.
46. Liu C, Shao Z. *Alcanivorax dieselolei* sp. nov., a novel alkane-degrading bacterium isolated from sea water and deep-sea sediment. International Journal of Systematic and Evolutionary Microbiology. 2005;55(3):1181-6.
47. Embar-Gopinath S, Butler R, Nowak B. Influence of salmonid gill bacteria on development and severity of amoebic gill disease. Diseases of aquatic organisms. 2005;67(1-2):55-60.
48. Embar-Gopinath S, Crosbie P, Nowak BF. Concentration effects of *Winogradskyella* sp. on the incidence and severity of amoebic gill disease. Diseases of aquatic organisms. 2006;73(1):43-7.
49. Riiser ES, Haverkamp TH, Borgan Ø, Jakobsen KS, Jentoft S, Star B. A single Vibrionales 16S rRNA oligotype dominates the intestinal microbiome in two geographically separated Atlantic cod populations. Frontiers in Microbiology. 2018;9:1561.
50. Svanevik CS, Lunestad BT. Characterisation of the microbiota of Atlantic mackerel (*Scomber scombrus*). International Journal of Food Microbiology. 2011;151(2):164-70.
51. Brown RM, Wiens GD, Salinas I. Analysis of the gut and gill microbiome of resistant and susceptible lines of rainbow trout (*Oncorhynchus mykiss*). Fish & shellfish immunology. 2019;86:497-506.
52. Udoye C. Analytical challenges and characterization of skin and gut microbiota of Atlantic salmon fry in a commercial smolt production facility (Master's thesis, NTNU).
53. Nikouli E, Meziti A, Antonopoulou E, Mente E, Kormas KA. Gut bacterial communities in geographically distant populations of farmed sea bream (*Sparus aurata*) and sea bass (*Dicentrarchus labrax*). Microorganisms. 2018;6(3):92.
54. Rasheeda MK, Rangamaran VR, Srinivasan S, Ramaiah SK, Gunasekaran R, Jaypal S, Gopal D, Ramalingam K. Comparative profiling of microbial community of three economically important fishes reared in sea cages under tropical offshore environment. Marine genomics. 2017 Aug 1;34:57-65.
55. Carlson JM, Leonard AB, Hyde ER, Petrosino JF, Primm TP. Microbiome disruption and recovery in the fish *Gambusia affinis* following exposure to broad-spectrum antibiotic. Infection and drug resistance. 2017;10:143.
56. Ciric M, Waite D, Draper J, Jones JB. Characterisation of gut microbiota of farmed Chinook salmon using metabarcoding. bioRxiv: The Preprint server for Biology. 2018.
57. Benedicenti, O., Secombes, C.J. and Collins, C., 2019. Effects of temperature on *Paramoeba perurans* growth in culture and the associated microbial community. Parasitology, 146(4), pp.533-542.
58. Hovda MB, Fontanillas R, McGurk C, Obach A, Rosnes JT. Seasonal variations in the intestinal microbiota of farmed Atlantic salmon (*Salmo salar* L.). Aquaculture Research. 2012;43(1):154-9.
59. Tan CK, Natrah I, Suyub IB, Edward MJ, Kaman N, Samsudin AA. Comparative study of gut microbiota in wild and captive Malaysian Mahseer (*Tor tambroides*). MicrobiologyOpen. 2019;8(5):e00734.
60. Tran NT, Zhang J, Xiong F, Wang GT, Li WX, Wu SG. Altered gut microbiota associated with intestinal disease in grass carp (*Ctenopharyngodon idellus*). World Journal of Microbiology and Biotechnology. 2018;34(6):71.
61. Gajardo K, Rodiles A, Kortner TM, Krogdahl Å, Bakke AM, Merrifield DL, Sørum H. A high-resolution map of the gut microbiota in Atlantic salmon (*Salmo salar*): a basis for comparative gut microbial research. Scientific Reports. 2016;6:30893.
62. Tacchi L, Bickerdike R, Douglas A, Secombes CJ, Martin SA. Transcriptomic responses to functional feeds in Atlantic salmon (*Salmo salar*). Fish & shellfish immunology. 2011;31(5):704-15.
63. Hou D, Huang Z, Zeng S, Liu J, Weng S, He J. Comparative analysis of the bacterial community compositions of the shrimp intestine, surrounding water and sediment. Journal of applied microbiology. 2018;125(3):792-9.
64. Liu H, Wang B, Hu X. Sediment bacterial communities are more complex in coastal shallow straits than in oceanic deep straits. Journal of Oceanology and Limnology. 2018;36(5):1643-54.
65. Valliappan K, Sun W, Li Z. Marine actinobacteria associated with marine organisms and their potentials in producing pharmaceutical natural products. Applied microbiology and biotechnology. 2014;98(17):7365-77.
66. Catalán N, Villasante A, Wacyk J, Ramírez C, Romero J. Fermented soybean meal increases lactic acid bacteria in gut microbiota of Atlantic Salmon (*Salmo salar*). Probiotics and antimicrobial proteins. 2018;10(3):566-76.
67. Chalupnicki M, Dittman D, Starliper CE, Iwanowicz DD. Efficacy of iodine for disinfection of lake sturgeon eggs from the St. Lawrence River, New York. North American Journal of Aquaculture. 2015;77(1):82-9.
68. Tuncer I, Bizsel N. Antibiotic resistance and phylogeny of bacterial isolates with biogeochemical analysis from sediments of Eastern Mediterranean Sea in association with environmental parameters. Journal of Clinical & Experimental Immunology. 2016;1(2):15-20.
69. Cortés-Lara S, Urdiain M, Mora-Ruiz M, Prieto L, Rosselló-Móra R. Prokaryotic microbiota in the digestive cavity of the jellyfish *Cotylorhiza tuberculata*. Systematic and applied microbiology. 2015;38(7):494-500.
70. Draghi A, Popov VL, Kahl MM, Stanton JB, Brown CC, Tsongalis GJ, West AB, Frasca S. Characterization of “*Candidatus* Piscichlamydia salmonis”(order Chlamydiales), a chlamydia-like bacterium associated with epitheliocystis in farmed Atlantic salmon (*Salmo salar*). Journal of clinical microbiology. 2004;42(11):5286-97.
71. MacPhail DP, Koppenstein R, Maciver SK, Paley R, Longshaw M, Henriquez FL. Vibrio species are predominantly intracellular within cultures of *Neoparamoeba perurans*, causative agent of Amoebic Gill Disease (AGD). Aquaculture. 2020, 736083.
72. Slinger J, Adams MB, Wynne JW. Bacteriomic profiling of branchial lesions induced by *Neoparamoeba perurans* challenge reveals commensal dysbiosis and an association with *Tenacibaculum dicentrarchi* in AGD-affected Atlantic salmon (*Salmo Salar* L.). Microorganisms. 2020, 8(8), 1189.

**Supplementary Table 2: Subset analysis of the Microbiome data**. Subsets obtained from “BVSTEP” routine. PERMANOVA of these subsets were also performed against sources of variations including Time, Sample Type (where applicable), and qPCR of *N. perurans*. For comparative purposes, PERMANOVA, using the full OTU table, data is also provided to highlight loss in variability with the subsets. Two cases were considered: Gill Longitudinal Analysis (white background) and Gill vs Mucus Cross-sectional Analysis (grey background). For each case, the abundance tables of the resulting subset of OTUs were obtained, normalised (log-relative normalisation), and then correlated (Kendall Correlation) with extrinsic parameters. The resulting p-values were then adjusted for multiple comparisons (Bonferroni-Hochberg procedure) for the OTUs found for the two cases and if significant, the correlation values (R) along with adjusted P-values are provided. The positive and negative correlations of these OTUs are then highlighted in different colours. For Gill vs Mucus Cross-sectional analysis, the correlations were performed separately for Gill and Mucus and together by collating the samples (as Gill + Mucus).

| **Gill Longitudinal Analysis** | | | | | |
| --- | --- | --- | --- | --- | --- |
| **Subset of top 1000 most abundant OTUs** | | **Correlation with full OTUs table** | **PERMANOVA full OTU table** | | |
|  |  |  | **Time and Sample Type** | | **Log10(qPCR of *N. perurans*)** |
|  |  |  | \| Time: R^2^ = 0.22 (p = 0.001 ***) \| \| --- \| \| Type: R^2^ = 0.04 (p = 0.001 ***) \| | | R^2^ = 0.05 (p = 0.001 ***) |
|  |  |  | **PERMANOVA subsets** | | |
| S1 | \| OTU_268 + OTU_145 + OTU_62 + OTU_154 +OTU_136 + OTU_347 + OTU_353 + OTU_335 + OTU_363 \| \| --- \| | 0.922 | \| R^2^ = 0.208 (p = 0.001 ***) \| \| --- \| | | R^2^ = 0.037 (p = 0.006 **) |
| S2 | \| OTU_268 + OTU_145 + OTU_62 + OTU_154 + OTU_136 + OTU_148 + OTU_347 + OTU_353 + OTU_335 + OTU_363 \| \| --- \| | 0.922 | \| R^2^ = 0.231 (p = 0.001 ***) \| \| --- \| \|  \| | | R^2^ = 0.028 (p = 0.015 *) |
| S3 | \| OTU_268 + OTU_145 + OTU_62 + OTU_154 + OTU_136 + OTU_353 + OTU_188 + OTU_335 + OTU_3156 + OTU_1011 \| \| --- \| | 0.922 | \| R^2^ = 0.224 (p = 0.001 ***) \| \| --- \| \|  \| | | R^2^ = 0.046 (p = 0.004 **) |
| S4 | OTU_268 + OTU_145 + OTU_62 + OTU_154 + OTU_136 + OTU_347 + OTU_353 | 0.915 | \| R^2^ = 0.224 (p = 0.001 ***) \| \| --- \| \|  \| | | R^2^ = 0.046 (p = 0.009 **) |
| S5 | OTU_268 + OTU_145 + OTU_62 + OTU_154 + OTU_347 + OTU_353 | 0.909 | \| R^2^ = 0.231 (p = 0.001 ***) \| \| --- \| \|  \| | | R^2^ = 0.047 (p = 0.005 **) |
| S6 | OTU_268 + OTU_145 + OTU_62 + OTU_154 + OTU_353 | 0.900 | \| R^2^ = 0.231 (p = 0.001 ***) \| \| --- \| \|  \| | | R^2^ = 0.047 (p = 0.006 **) |
| S7 | OTU_268 + OTU_145 + OTU_62 + OTU_154 | 0.889 | \| R^2^ = 0.231 (p = 0.001 ***) \| \| --- \| \|  \| | | R^2^ = 0.048 (p = 0.008 **) |
| S8 | OTU_268 + OTU_62 + OTU_154 | 0.873 | \| R^2^ = 0.238 (p = 0.001 ***) \| \| --- \| \|  \| | | R^2^ = 0.051 (p = 0.004 **) |
| S9 | OTU_268 + OTU_154 | 0.832 | \| R^2^ = 0.427 (p = 0.93) \| \| --- \| \|  \| | | R^2^ = 0.061 (p = 0.937) |
| **Correlations with environmental factors and fish features** | | | | | |
| OTU_268: Lactobacillaceae;Lactobacillus  OTU_145: Erysipelotrichaceae;Turicibacter  OTU_62: Xanthomonadaceae;Stenotrophomonas  OTU_154: Lactobacillaceae;Lactobacillus  OTU_136: Bifidobacterium;Bifidobacterium sp. AGR2158  OTU_347: Erysipelotrichaceae;Allobaculum  OTU_353: Clostridiaceae1;Clostridiumsensustricto 1  OTU_335: Lactobacillaceae;Lactobacillus  OTU_363: Xanthomonadales;Xanthomonadaceae;Stenotrophomonas  OTU_148: Propionibacteriaceae;Propionibacterium | | | | OTU_363: Clarity (previous month): R = -0.292 (Adj.p = 0.013 *) [Gill] | |
| **Gill vs Mucus Cross-sectional Analysis** | | | | | |
| **Subset of top 1000 most abundant OTUs** | | **Correlation with full OTUs table** | **PERMANOVA full OTU table** | | |
|  |  |  | **Time and Sample Type** | | **Log10 (qPCR of *N. perurans*)** |
|  |  |  | \| Time: R^2^ = 0.22 (p = 0.001 ***) \| \| --- \| \| Type: R^2^ = 0.04 (p = 0.001 ***) \| | | R^2^ = 0.05 (p = 0.001 ***) |
|  |  |  | **PERMANOVA Subsets** | | |
| S1 | \| OTU_268 + OTU_145 + OTU_62 + OTU_154 + \| \| --- \| \| OTU_136 + OTU_148 + OTU_353 + OTU_796 + \| \| OTU_188 + OTU_335 + OTU_3156 + OTU_1011 \| | 0.914 | \| Time: R^2^ = 0.14 (p = 0.001 ***) \| \| --- \| \| Type: R^2^ = 0.08 (p = 0.001 ***) \| | | R^2^ = 0.024 (p = 0.023 *) |
| S2 | \| OTU_268 + OTU_145 + OTU_62 + OTU_154 + \| \| --- \| \| OTU_136 + OTU_353 + OTU_796 + OTU_188 + \| \| OTU_335 + OTU_3156 + OTU_1011 \| | 0.912 | \| Time: R^2^ = 0.14 (p = 0.001 ***) \| \| --- \| \| Type: R^2^ = 0.09 (p = 0.001 ***) \| | | R^2^ = 0.029 (p = 0.014 *) |
| S3 | \| OTU_268 + OTU_145 + OTU_62 + OTU_154 + \| \| --- \| \| OTU_136 + OTU_353 + OTU_188 + OTU_335 + \| \| OTU_3156 + OTU_1011 \| | 0.912 | \| Time: R^2^ = 0.15 (p = 0.001 ***) \| \| --- \| \| Type: R^2^ = 0.09 (p = 0.001 ***) \| | | R^2^ = 0.030 (p = 0.014 *) |
| S4 | OTU_268 + OTU_145 + OTU_62 + OTU_154 + OTU_136 + OTU_353 + OTU_188 + OTU_335 + OTU_1011 | 0.903 | \| Time: R^2^ = 0.15 (p = 0.001 ***) \| \| --- \| \| Type: R^2^ = 0.09 (p = 0.001 ***) \| | | R^2^ = 0.30 (p = 0.007 **) |
| S5 | OTU_268 + OTU_145 + OTU_62 + OTU_154 + OTU_136 + OTU_353 + OTU_188 + OTU_335 | 0.896 | \| Time: R^2^ = 0.095 (p = 0.002 **) \| \| --- \| \| Type: R^2^ = 0.089 (p = 0.001 ***) \| | | R^2^ = 0.032 (p = 0.011 *) |
| S6 | OTU_268 + OTU_145 + OTU_62 + OTU_154 + OTU_136 + OTU_353 + OTU_188 | 0.883 | \| Time: R^2^ = 0.094 (p = 0.001 ***) \| \| --- \| \| Type: R^2^ = 0.089 (p = 0.001 ***) \| | | R^2^ = 0.032 (p = 0.014 *) |
| S7 | OTU_268 + OTU_62 + OTU_154 + OTU_136 + OTU_353 + OTU_188 | 0.865 | \| Time: R^2^ = 0.097 (p = 0.001 ***) \| \| --- \| \| Type: R^2^ = 0.089 (p = 0.001 ***) \| | | R^2^ = 0.033 (p = 0.011 *) |
| S8 | OTU_268 + OTU_62 + OTU_136 + OTU_353 + OTU_188 | 0.848 | \| Time: R^2^ = 0.100 (p = 0.001 ***) \| \| --- \| \| Type: R^2^ = 0.090 (p = 0.001 ***) \| | | R^2^ = 0.35 (p = 0.006 **) |
| S9 | OTU_268 + OTU_62 + OTU_136 + OTU_188 | 0.825 | \| Time: R^2^ = 0.100 (p = 0.001 ***) \| \| --- \| \| Type: R^2^ = 0.090 (p = 0.001 ***) \| | | R^2^ = 0.035 (p = 0.012 *) |
| S10 | OTU_268 + OTU_136 + OTU_188 | 0.770 | \| Time: R^2^ = 0.134 (p = 0.076) \| \| --- \| \| Type: R^2^ = 0.048 (p = 0.064) \| | | R^2^ = 0.050 (p = 0.072) |
| S11 | OTU_268 + OTU_62 + OTU_188 | 0.763 | \| Time: R^2^ = 0.106 (p = 0.001 ***) \| \| --- \| \| Type: R^2^ = 0.090 (p = 0.001 ***) \| | | R^2^ = 0.038 (p = 0.004 **) |
| S12 | OTU_268 + OTU_62 | 0.702 | \| Time: R^2^ = 0.090 (p = 0.003 **) \| \| --- \| \| Type: R^2^ = 0.114 (p = 0.001 ***) \| | | R^2^ = 0.041 (p = 0.008 **) |
| **Correlations with environmental factors and fish features** | | | | | |
| OTU_268: Lactobacillaceae;Lactobacillus  OTU_145: Erysipelotrichaceae;Turicibacter  OTU_62: Xanthomonadaceae;Stenotrophomonas  OTU_154: Lactobacillaceae;Lactobacillus  OTU_136: Bifidobacterium;Bifidobacterium sp. AGR2158  OTU_148: Propionibacteriaceae;Propionibacterium  OTU_353: Clostridiaceae1;Clostridiumsensustricto 1  OTU_796: Lactobacillaceae;Lactobacillus  OTU_188: Bifidobacteriaceae;Gardnerella  OTU_335: Lactobacillaceae;Lactobacillus  OTU_3156: Acidimicrobiaceae;Illumatobacter  OTU_1011: Comamonadaceae;Delftia | | OTU_148: Temperature (previous month): R = 0.288 (Adj.p = 0.004 **) [Gill]  OTU_188: Temperature (previous month): R = 0.50 (Adj.p<0.001 ***) [Gill]  OTU_188: Salinity (previous month): R = -0.459 (Adj.p<0.001 ***) [Gill]  OTU_188: Temperature (previous month): R = 0.50 (Adj.p<0.001 ***) [Gill+Mucus]  OTU_188: Salinity (previous month): R = -0.459 (Adj.p<0.001 ***) [Gill+Mucus]  OTU_188: Temperature (same day): R = 0.484 (Adj.p<0.001 ***) [Gill]  OTU_188: Clarity (same day): R = -0.319 (Adj.p = 0.033 *) [Gill]  OTU_188: Temperature (same day): R = 0.379 (Adj.p<0.001 ***) [Gill+Mucus]  OTU_188: Clarity (same day): R = -0.295 (Adj.p = 0.016 *) [Gill+Mucus]  OTU_188: AGD score of the fish: R = 0.297(Adj.p = 0.037 *) [Gill]  OTU_188: Weight of the fish: R = -0.240 (Adj.p = 0.018 *) [Gill+Mucus]  OTU_188: AGD score of the fish: R = 0.268 (Adj.p = 0.015 *) [Gill+Mucus]  OTU_3156: Oxygen Levels (previous month): R = -0.295 (Adj.p = 0.019 *) [Gill]  OTU_3156: Oxygen Levels (previous month): R = -0.242 (Adj.p = 0.032 *) [Gill+Mucus]  OTU_3156: Temperature (same day): R = -0.227 (Adj.p = 0.036 *) [Gill+Mucus]  OTU_3156: Oxygen Levels (same day): R = -0.267 (Adj.p = 0.025 *) [Gill+Mucus]  OTU_3156: Temperature (same day): R = -0.270 (Adj.p = 0.027 *) [Gill]  OTU_1011: Temperature (previous month): R = 0.626 (Adj.p<0.001 ***) [Gill]  OTU_1011: Salinity (previous month): R = -0.396 (Adj.p<0.001 ***) [Gill]  OTU_1011: Oxygen Levels (previous month): R = 0.255 (Adj.p = 0.019 *) [Gill]  OTU_1011: Temperature (previous month): R = 0.395 (Adj.p<0.001 ***) [Gill+Mucus]  OTU_1011: Salinity (previous month): R = -0.310 (Adj.p<0.001 ***) [Gill+Mucus]  OTU_1011: Oxygen Levels (previous month): R = 0.257 (Adj.p = 0.006 **) [Gill+Mucus]  OTU_1011: Temperature (same day): R = 0.419 (Adj.p<0.001 ***) [Gill+Mucus]  OTU_1011: Temperature (same day): R = 0.568 (Adj.p<0.001 ***) [Gill] | | | |

**Supplementary Table 3. Subset regression to link *N. perurans* to explanatory data.** The linear regression was fitted to the form, Y_i_ = β _0_ + β_1_ X1_i_ + β_2_ X2_i_ + β_3_ X3_i_ + ε_i_, where Y_i_ is the Log10 (qPCR of *N. perurans*). Using leaps package in the regression model, the subsets of explanatory variables X_i_ were permuted, and report the top 20 subsets ranked by adjusted R^2^. Furthermore, cross-validation for linear regression was performed using CVlm() from R’s DAAG package. Ten folds in the function were used whereby data are randomly assigned to the number of folds, each fold is removed, and in turn, the remaining data issued to re-fit the regression model and to predict at the deleted observations. Mean Squared Error (MSE) between cross-validated prediction and the predicted values using all observations is thus reported in the table. Predictors with positive β-coefficients (that are significant) and are causing *N. perurans* to increase are shaded as red, and those with negative β-coefficients (that are significant) and are causing *N. perurans* to decrease are shaded as blue. If the explanatory variable is not selected in the subset regression, the cell is empty. A consistent red or blue pattern (in column) for a predictor frequently selected in a regression model can serve as a clue for its importance. Note that of all the explanatory variables considered, Type (Gill and Mucus) and Time (point1, point2, point3, point4, point5, point6) are categorical variables were internally supplied to the regression model in R as factors. Also, in R when a categorical variable is considered, it is expanded to the number of factors (presence/absence) in that variable each with the iron β-coefficients. If Type:Mucus comes out to be significant and has a positive β-coefficient then it is read as more likely the sample comes from Mucus, more likely it increases *N*. *perurans*. In subset regression analysis, Type:Gill, Time:point1,Time:point5, and Time:point6 were not selected in any of the models and therefore are dropped from the list below.


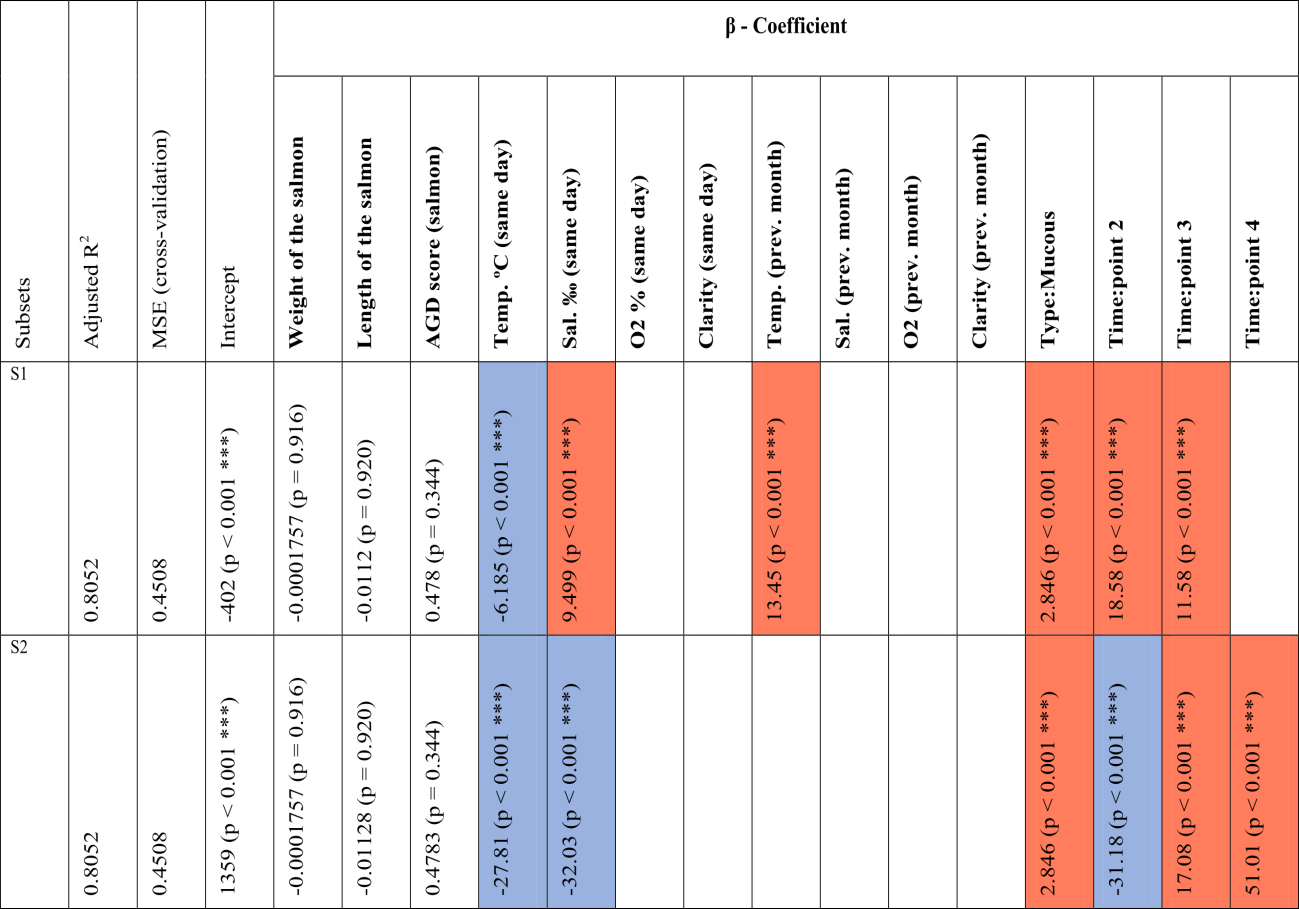

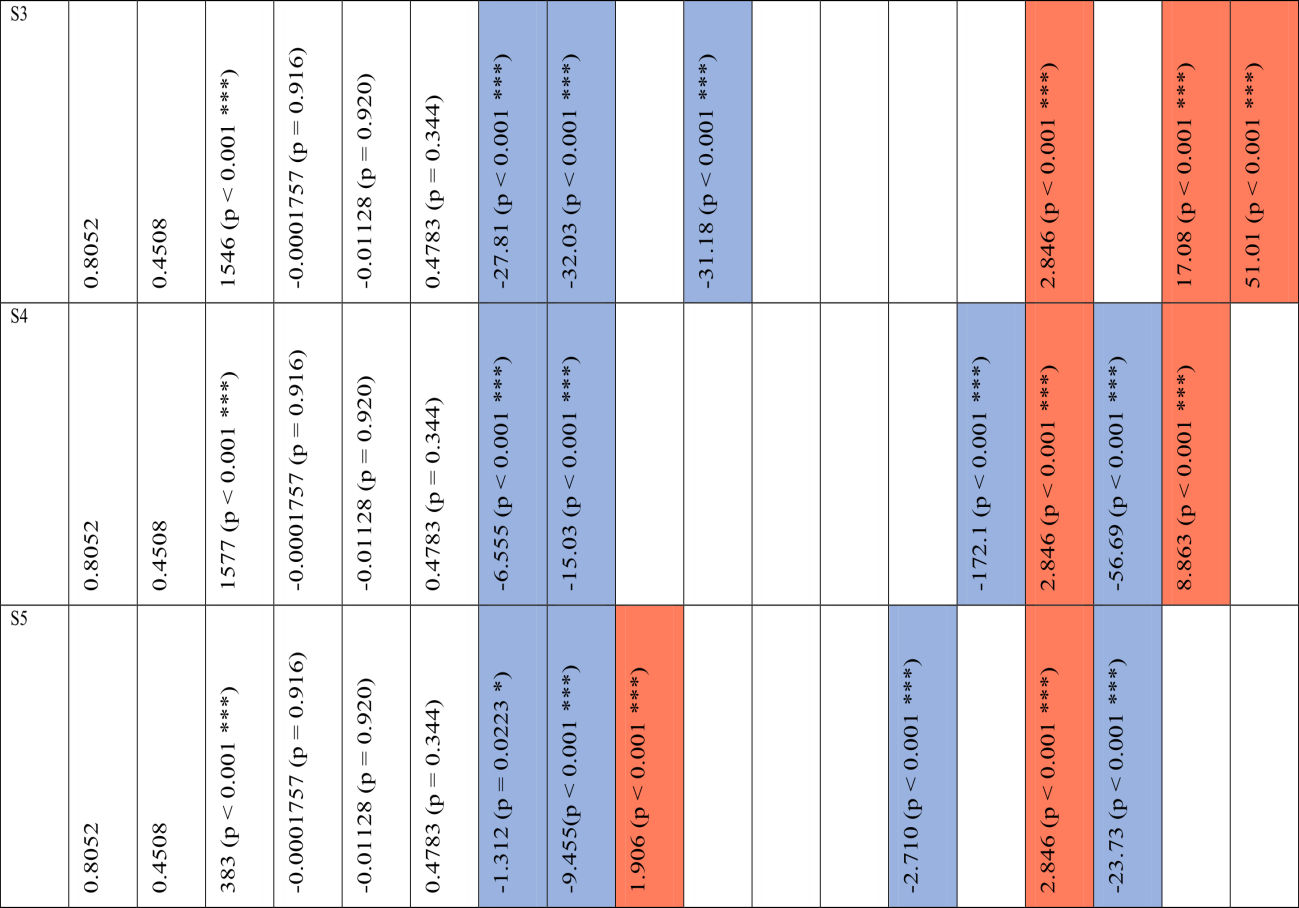


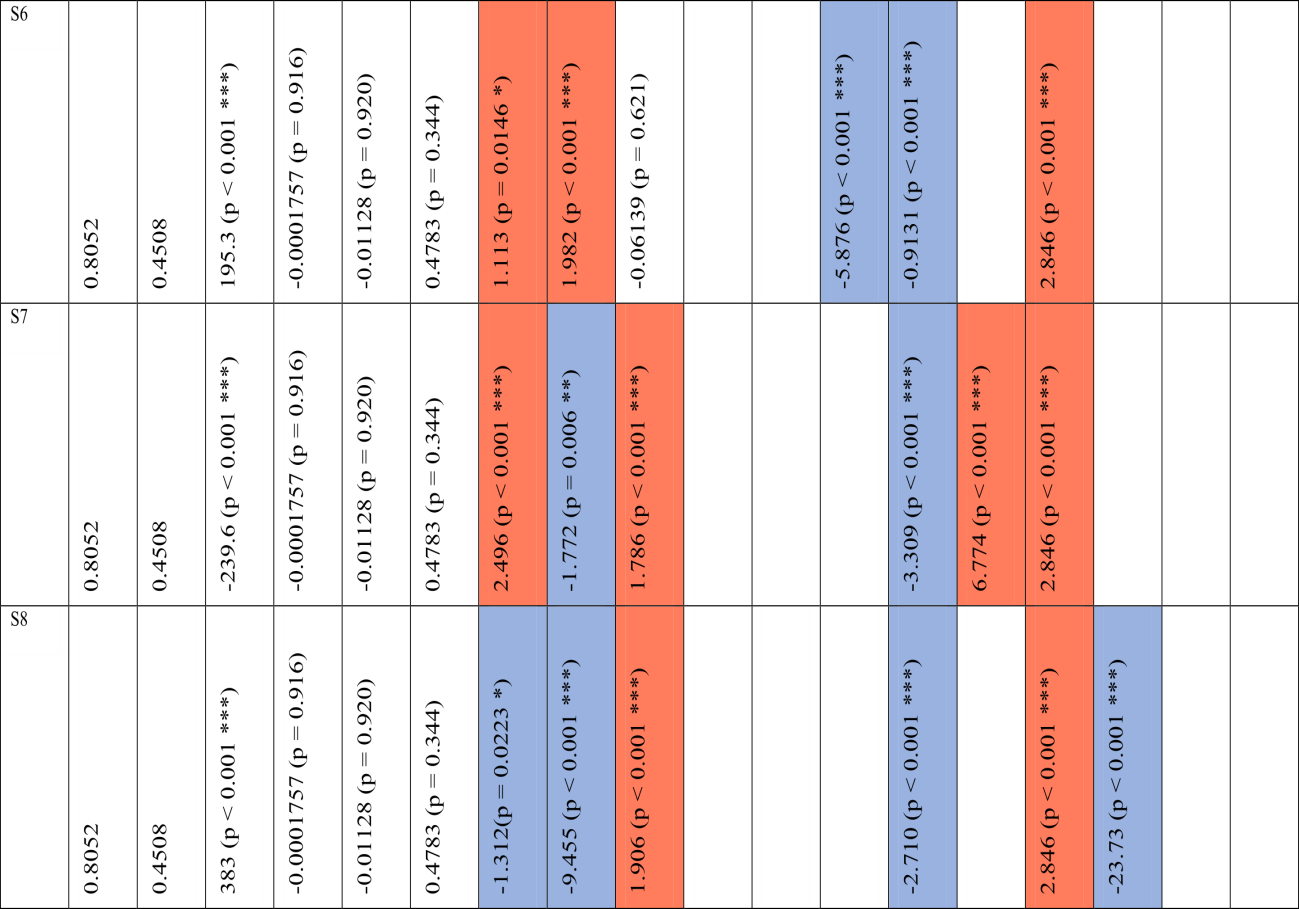


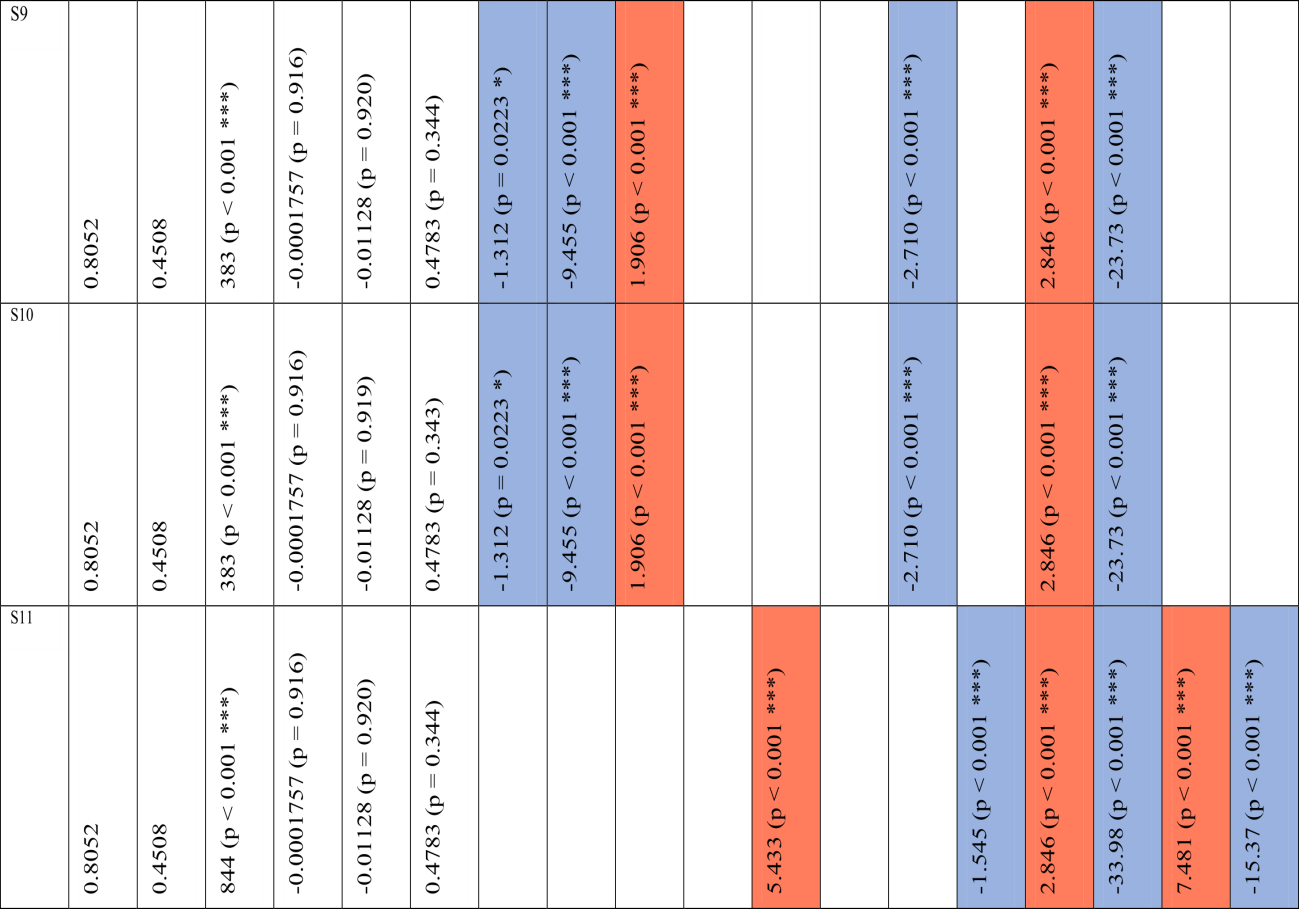


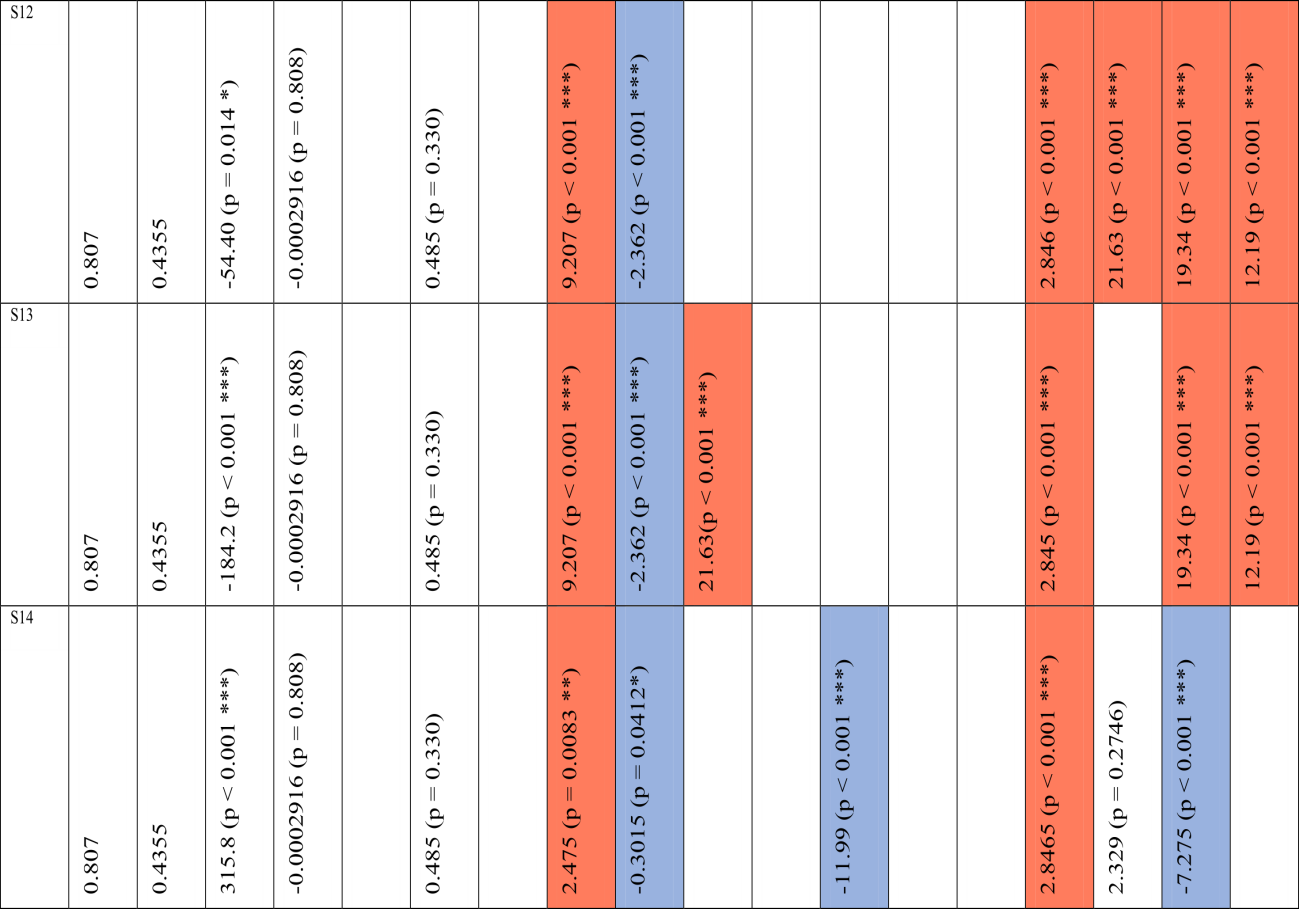


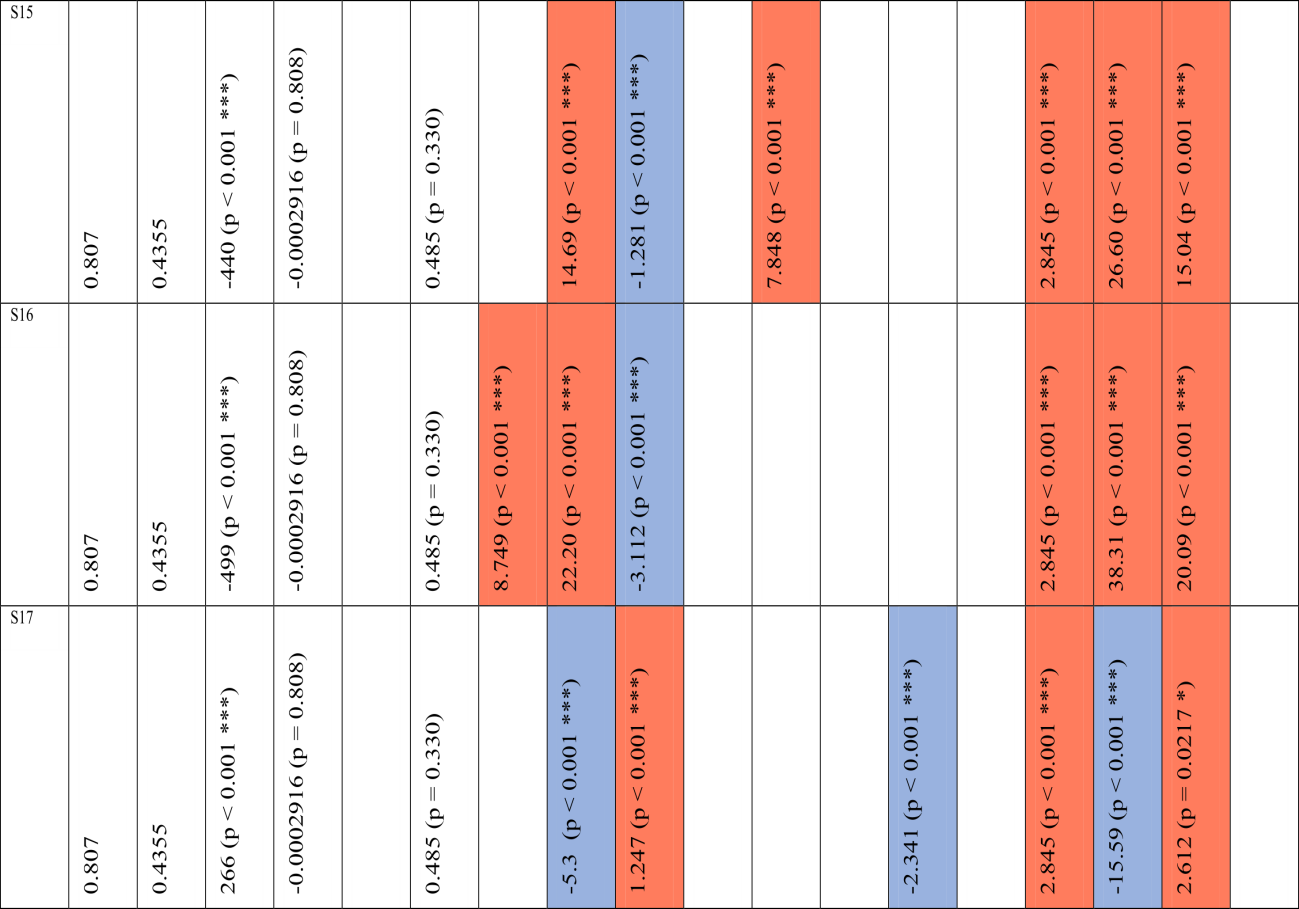


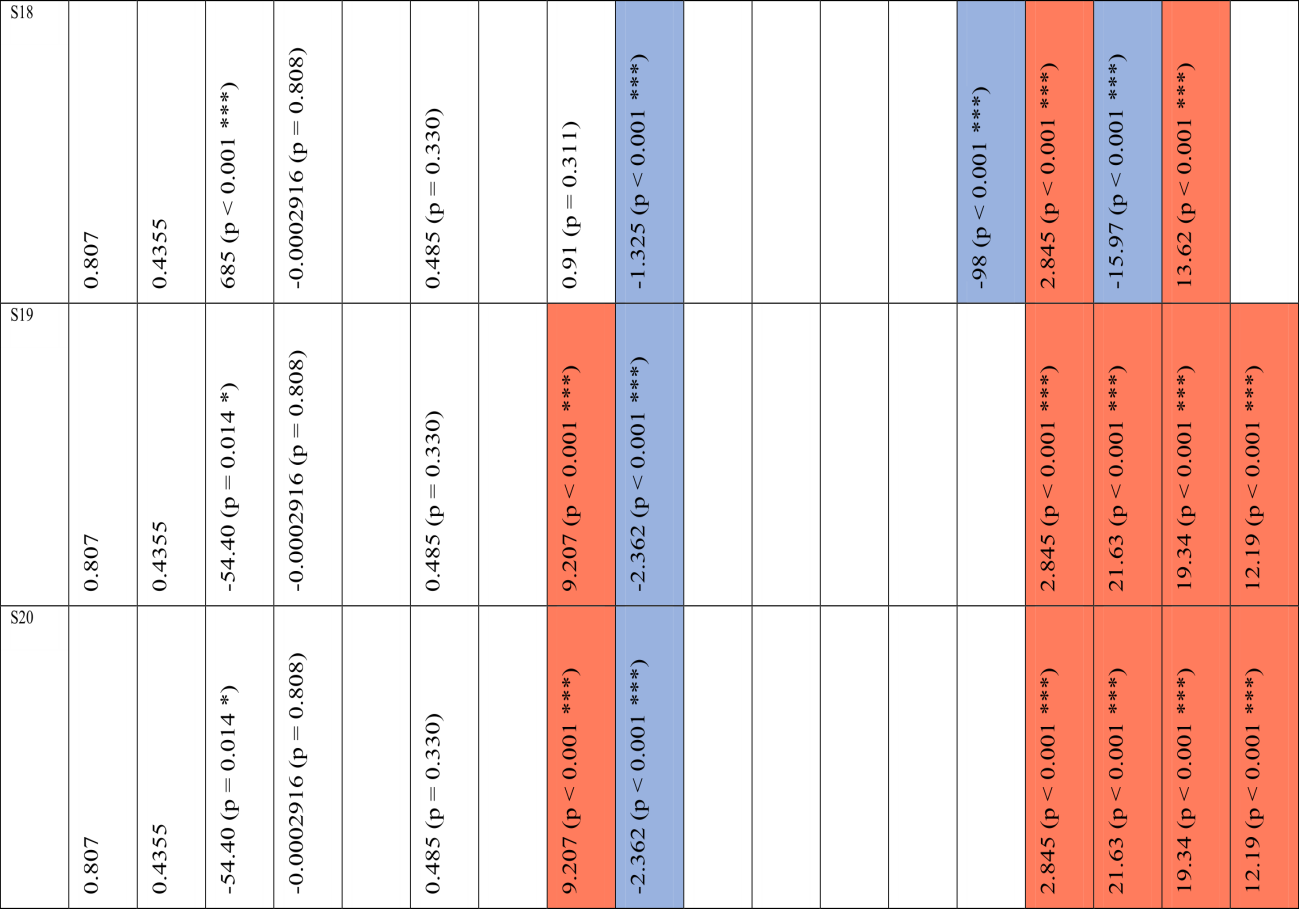


**Supplementary Table 4.** Mean and standard deviation (in brackets) values of the weight (g), length (cm), condition factor and gill scores of the 5 salmon from each of the cages in each timepoint. Each timepoint includes the date and the events in each of the important dates of the sampling campaign.

| **Date** | **Timepoint** | **Event** | **Cage ID** | **Weight (g)** | **Length (cm)** | **Condition Factor** | **Gill scores** |
| --- | --- | --- | --- | --- | --- | --- | --- |
| 05 May 2017 | T0 | Pre-smolted salmon sampling | 44 | 82.2 (7.5) | 20.2 (0.6) | 0.99  (0.04) | 0  (0) |
|  |  |  | 58 | 79.4 (6.6) | 19.7 (0.7) | 1.03  (0.06) | 0  (0) |
|  |  |  | 75 | 67.0 (11.0) | 18.5 (0.7) | 1.02  (0.06) | 0  (0) |
| 26 May 2017 | T1 | Post-smolted salmon sampling | 1 | 144.5 (42.9) | 22.8 (1.8) | 1.18  (0.1) | 0  (0) |
|  |  |  | 4 | 80.4 (6.0) | 19.5 (0.3) | 1.08  (0.05) | 0  (0) |
|  |  |  | 6 | 75.8 (11.9) | 19.4 (0.7) | 1.02  (0.12) | 0  (0) |
| 22 June 2017 | T2 | Post-smolted salmon sampling | 1 | 260.2 (68.8) | 28.3 (3.2) | 1.14  (0.25) | 0  (0) |
|  |  |  | 4 | 137.3 (25.8) | 24.1 (1.4) | 0.96  (0.02) | 0  (0) |
|  |  |  | 6 | 135.4 (41.7) | 23.9 (2.4) | 0.95  (0.08) | 0  (0) |
| 11 July 2017 | T3 | AGD-affected Post-smolted salmon sampling | 1 | 267.7  (66.8) | 29.6  (1.6) | 1.01  (0.1) | 1.6  (0.5) |
|  |  |  | 4 | 105.9  (20.9) | 23.0  (1.1) | 0.85  (0.03) | 1.0  (0.7) |
|  |  |  | 6 | 117.4  (50.2) | 23.4 (3.4) | 0.86  (0.06) | 1.4  (0.5) |
| 18 July 2017 |  | First freshwater bath | 1 and 4 |  |  |  |  |
| 19 July 2017 |  | First freshwater bath | 6 |  |  |  |  |
| 10 August 2017 | T4 | AGD-affected Post-smolted salmon sampling | 1 | 295.0  (30.0) | 29.8  (1.3) | 1.1  (0.08) | 1.6  (0.5) |
|  |  |  | 4 | 158.4  (29.7) | 26.3  (1.1) | 0.86  (0.05) | 1.8  (0.4) |
|  |  |  | 6 | 158.4  (29.7) | 26.3  (1.1) | 0.85  (0.07) | 1.4  (0.5) |
| 25 September 2017 | T5 | AGD-affected Post-smolted salmon sampling | 1 | 347.7  (122.5) | 33.2  (1.8) | 0.91  (0.14) | 0.4  (0.2) |
|  |  |  | 4 | 181.6  (58.0) | 26.9  (2.1) | 0.9  (0.09) | 1.2  (0.8) |
|  |  |  | 6 | 274.8 (199.5) | 29.8  (4.6) | 0.91  (0.18) | 1.0  (1.0) |
| 09 October 2017 |  | Second freshwater bath | 1 and 4 |  |  |  |  |
| 10 October 2017 |  | Second freshwater bath | 6 |  |  |  |  |
| 26 October 2017 | T6 | AGD-affected Post-smolted salmon sampling | 1 | 1103.7  (574.4) | 38.1  (3.4) | 1.85  (0.45) | 2.0  (0.2) |
|  |  |  | 4 | 657.1  (308.1) | 36.3  (2.8) | 1.31  (0.37) | 1.0  (0.0) |
|  |  |  | 6 | 781.1  (572.0) | 34.6  (4.7) | 1.6  (0.57) | 1.2  (0.4) |
